# Genomic epidemiology of carbapenemase-producing *Klebsiella pneumoniae* circulating in a Chilean tertiary-care hospital (2021–2022): Molecular characterization, resistance-virulence convergence, and clinical associations

**DOI:** 10.15698/mic2026.05.876

**Published:** 2026-05-21

**Authors:** Gustavo Araya, Alhejandra Álvarez, Carolina Arellano, Rodrigo Bravo, Abraham Gajardo, Boris Barrera, Francisco Silva, Roberto M. Vidal

**Affiliations:** 1 Núcleo de Microbiología, Instituto de Ciencias Biomédicas (ICBM), Facultad de Medicina, Universidad de Chile, Chile; 2 Unidad de Pacientes Críticos, Departamento de Enfermería, Hospital Clínico Universidad de Chile, Chile; 3 Escuela de Medicina, Facultad de Medicina, Universidad de Chile, Chile; 4 Unidad de Cuidados Intensivos, Departamento de Medicina Interna, Hospital Clínico Universidad de Chile, Chile; 5 Núcleo de Fisiología, Biofísica y Fisiopatología, Instituto de Ciencias Biomédicas (ICBM), Facultad de Medicina, Universidad de Chile, Chile; 6 Unidad de Microbiología, Laboratorio Clínico, Hospital Clínico Universidad de Chile, Chile

**Keywords:** *Klebsiella pneumoniae*, carbapenem resistance, virulence factors, multidrug-resistant bacteria, whole genome sequencing (WGS)

## Abstract

*Klebsiella pneumoniae* (Kp) is a Gram-negative bacillus responsible for approximately 10% of nosocomial bacterial infections and one-third of Gram-negative bacterial infections in hospitalized patients. The rise of multidrug-resistant and hypervirulent strains makes it a significant public health issue. This study characterized carbapenem-resistant Kp (CR-Kp) strains isolated at the Hospital Clínico Universidad de Chile (HCUCH) 2021–2022 and explored associations with clinical characteristics. 45 CR-Kp strains from 29 patients in critical care units were analyzed. Mass spectrometry was used for species identification, and antimicrobial susceptibility was assessed by Kirby-Bauer disk diffusion. Clonality was determined using pulsed-field gel electrophoresis (PFGE), and multiplex PCR detected resistance and virulence genes. Clonal strains underwent whole-genome sequencing. PCR revealed the high prevalence of carbapenemase genes and extended-spectrum β -lactamases. PFGE identified nine clones, corresponding to sequence types ST25, ST45, ST307, and ST1161. Frequent virulence factors included siderophores and adhesins, while the capsular serotype K2 was present in 44% of isolates. No classical hypervirulence markers were detected. The presence of *blaKPC* correlated with more extended hospitalization. These findings reveal convergence between multidrug resistance and adaptive virulence traits rather than classical hypervirulence, highlighting evolving pathogenic strategies in high-risk CR-Kp clones circulating in Chile and emphasizing the need for enhanced molecular surveillance and infection protocols in critical care settings.

## INTRODUCTION

The antibiotic era has been marked by significant discoveries of antimicrobial agents, accompanied by a steady emergence of bacterial resistance mechanisms. Antibiotic resistance remains a growing concern due to the slow advancement of novel drugs, with an estimated annual economic impact exceeding USD$105 billion 1. Failure to address antibiotic resistance could lead to a global population decline of 11 to 444 million people by 2050 2, with mortality rates comparable to those of cancer 3, particularly affecting low-income countries where resistance rates are highest 4. β -lactamase production is the primary resistance mechanism among Gram-negative bacteria to β -lactams 5. Extended-spectrum β -lactamases (ESBLs), particularly in *Klebsiella pneumoniae* and *Escherichia coli*, are associated with limited therapeutic options and elevated mortality rates 6. Carbapenems, bactericidal β -lactams capable of resisting ESBL-mediated hydrolysis, became the treatment of choice against ESBL-producing infections 1, 6, 7. In 2017 and again in 2024, the World Health Organization (WHO) designated carbapenem-resistant Enterobacterales (CRE) as a critical priority group for new antibiotic development 8. *K. pneumoniae* is frequently among the leading species in healthcare-associated infections, although the relative contribution varies markedly across regions, institutions, and study periods 9. Similarly, the reported prevalence of carbapenem-resistant *K. pneumoniae* (CR-Kp) colonization in ICU units varies greatly across settings and surveillance strategies, and can reach high levels in some cohorts 10. Invasive CR-Kp infections are associated with substantial morbidity and mortality, with estimates differing based on infection site and patient population, including reports from Europe 11. Resistance mechanisms in CR-Kp include carbapenemase production (class A [KPC, GES], B [IMP, VIM, NDM], and D [OXA-48]) 12, 13, alongside porin alterations and efflux pumps 14. Mobile genetic elements such as plasmids, transposons, and integrons facilitate the horizontal transmission of resistance genes, including *bla*KPC, *bla*NDM, and *bla*OXA 15–18. Clonal expansion of high-risk clones like ST258 and ST307 further accelerates the spread of resistance globally 19–22. In addition to resistance, *K. pneumoniae* exhibits multiple virulence factors, including capsular polysaccharides, fimbrial adhesins, lipopolysaccharides, and siderophores 23–27. Classical *K. pneumoniae* (cKp) strains mainly cause healthcare-associated infections, while hypervirulent strains (hvKp) are responsible for invasive diseases 28, 29. Alarmingly, hypervirulence and multidrug resistance convergence is being observed with increasing frequency 30. The first CR-Kp case in Chile was reported in 2012 31, and national surveillance has documented increasing detection of carbapenemase-producing Enterobacterales since the mid-2010s, with *blaKPC* frequently identified 32, 33. Chilean surveillance reports indicate progressive expansion of blaKPC-carrying lineages since 2014, particularly in critical-care settings, underscoring the need for local genomic studies to contextualize regional transmission patterns. Expanded genomic surveillance is urgently required to clarify local epidemiology, strengthen infection control strategies, and guide informed clinical management.

**Figure 1:**
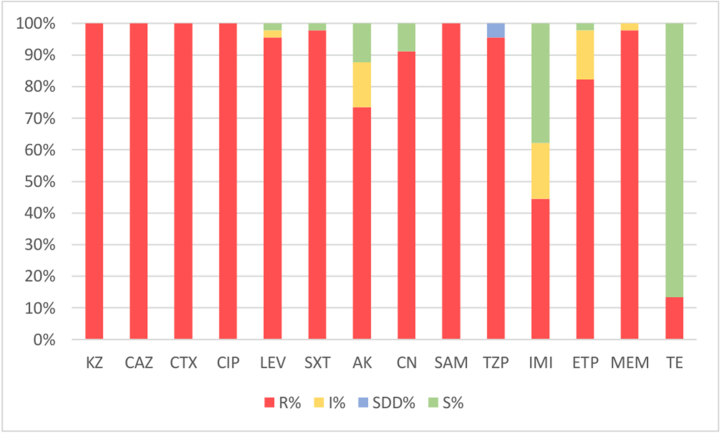
Antibiotic susceptibility profile of *K. pneumoniae* isolates. Y-axis: percentage of resistant strains. X-axis: antibiotics tested. Abbreviations: susceptible (S), intermediate susceptibility (I), susceptible dose-dependent (SDD), and resistant (R); KZ: Cefazolin, CAZ: Ceftazidime, CTX: Cefotaxime, CIP: Ciprofloxacin, LEV: Levofloxacin, SXT: Trimethoprim/Sulfamethoxazole, AK: Amikacin, CN: Gentamicin, SAM: Ampicillin/Sulbactam, TZP: Piperacillin/Tazobactam, IMI: Imipenem, ETP: Ertapenem, MEM: Meropenem, TE: Tetracycline.

## RESULTS

**Figure 2:**
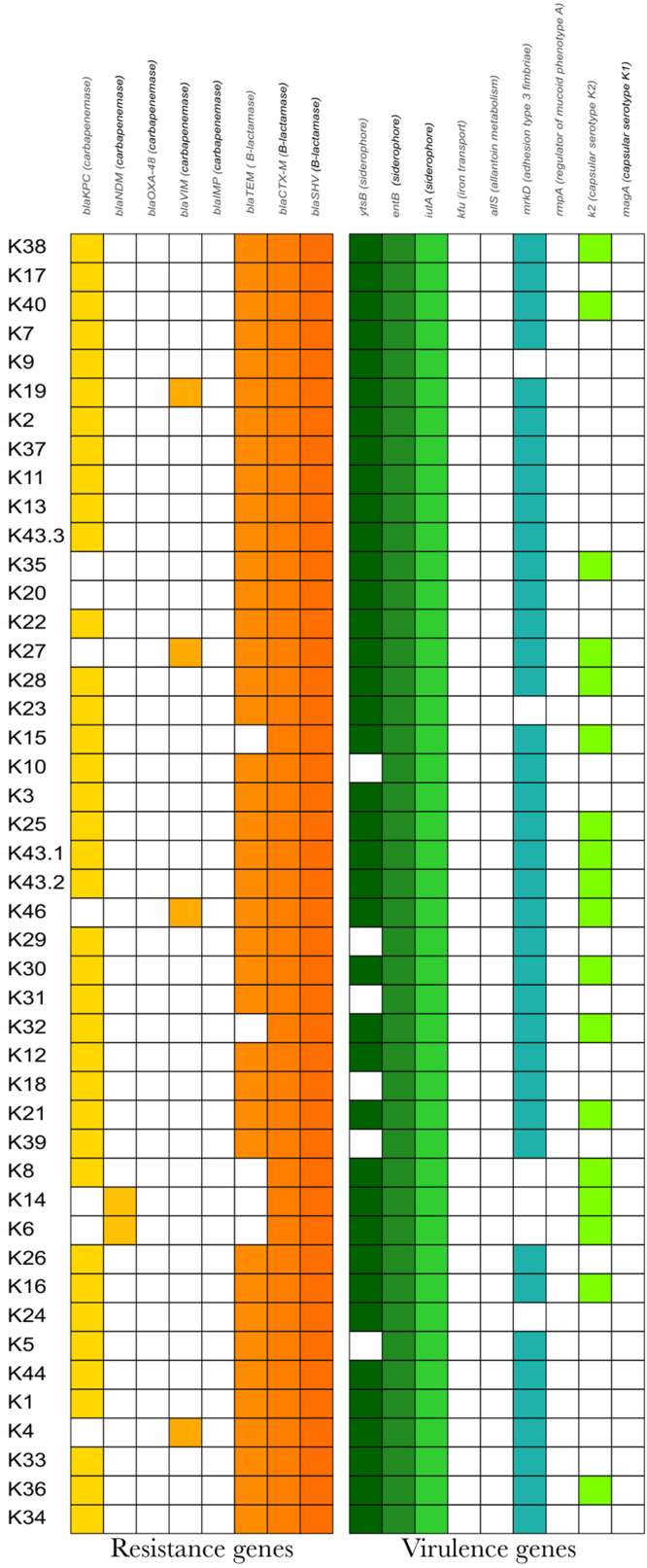
Summary of molecular features detected by PCR. Molecular characteristics of *K. pneumoniae* strains included in this study were assessed by PCR. **Yellow**: presence of resistance genes. **Green**: presence of virulence factors. **White**: absence.

**Figure 3:**
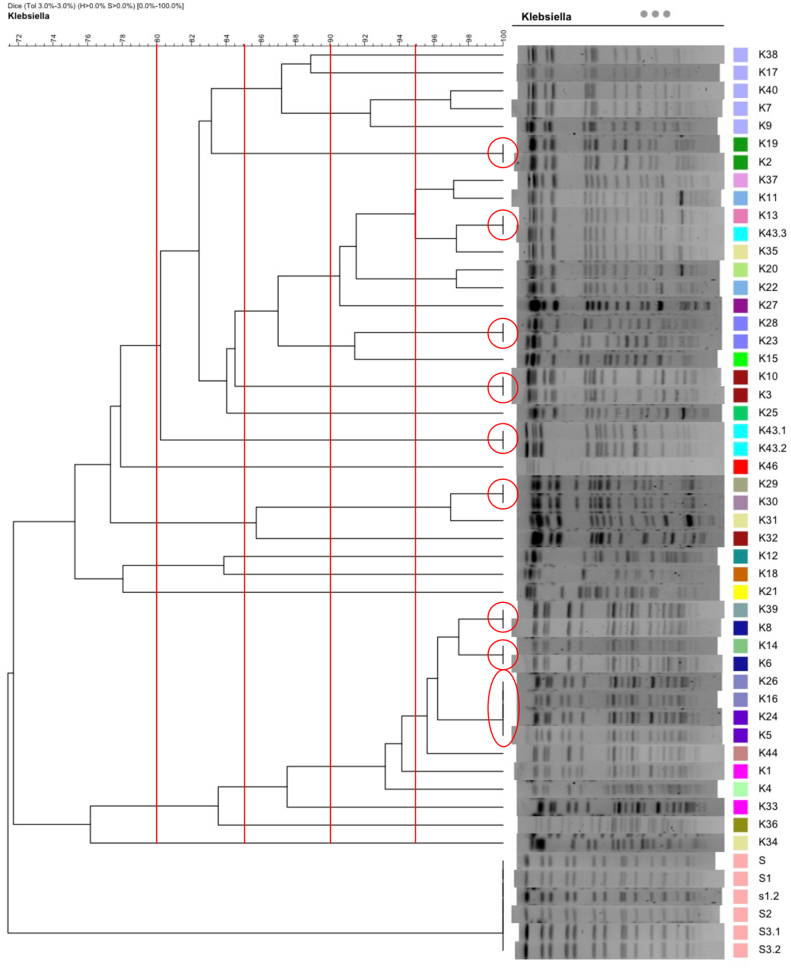
PFGE dendrogram of CR-Kp isolates. Dendrogram generated using the Dice similarity coefficient and the UPGMA method, with a 3% tolerance. The upper axis shows the percentage of similarity. Banding patterns for each strain are displayed. Red lines indicate the four discrimination thresholds used (80%, 85%, 90%, and 95% similarity). Red circles highlight clonal strains. **K**: study strains; **S**: control strains (*Salmonella* Braenderup).

Phenotypic antibiotic susceptibility testing revealed a high level of resistance among the *Klebsiella pneumoniae* isolates. All the strains demonstrated resistance to at least one carbapenem. Universal resistance (100%) was observed for cefazolin, ceftazidime, cefotaxime, ciprofloxacin, and ampicillin/sulbactam. High resistance was also noted for sulfamethoxazole-trimethoprim (98%) and gentamicin (91%). Tetracycline exhibited the highest sensitivity rate (87%), followed by imipenem (38%) and amikacin (13%). To facilitate visualization, the antibiotic resistance distribution was illustrated graphically (Figure 1), underscoring the high prevalence of resistance across several antimicrobial classes. Extended-spectrum β -lactamase (ESBL) production was detected phenotypically in 98% of isolates. In disk diffusion comparisons, 89% showed a ≥ 5 mm increase in zone diameter with ceftazidime/clavulanic acid and 71% with cefotaxime/clavulanic acid, confirming ESBL activity. Following the Pan American Health Organization criteria, 6.6% of isolates were classified as multidrug-resistant (MDR), 86.6% as extensively drug-resistant (XDR), and 6.6% as pandrug-resistant (PDR). PCR assays detected carbapenemase genes in 96% of isolates, with *bla*KPC being the most prevalent (84%), followed by *bla*VIM (9%) and *bla*NDM (4%) (Figure S1). No isolates carried *blaOXA-48* or *blaIMP*. Two isolates (2/45; 4%) were reported as carbapenemase-positive by routine phenotypic testing performed at the clinical microbiology laboratory (CarbaNP and immunochromatography); however, they were negative for the carbapenemase genes included in our multiplex PCR panel. No additional molecular characterization was performed for these isolates beyond the assays reported here. The analysis of β -lactamase genes revealed a 100% prevalence of *blaCTX-M* and *blaSHV*, with 89% also carrying *blaTEM* (Figure S2). The almost universal presence of *blaCTX-M* correlates with the high frequency of the ESBL phenotype. Virulence gene screening showed a high prevalence of siderophore-associated genes *entB* and *iutA* (100%), *ytsB* (91%), as well as the type 3 fimbrial adhesin gene *mrkD* (87%), and the capsular serotype K2 gene *k2* (44%) (Figure S3). The detection of multiple siderophores suggests enhanced iron acquisition capabilities, potentially increasing pathogenicity. A detailed molecular characterization chart summarized the presence of resistance and virulence genes found using PCR (Figure 2). Pulsed-field gel electrophoresis (PFGE) revealed nine clonal clusters among 20 isolates (Figure 3). Using multiple discriminatory thresholds (80%, 85%, 90%, and 95%), the isolates were classified into pulse groups and pulsotypes, reflecting substantial PFGE pattern diversity and typing discriminatory power, as indicated by Simpson’s diversity indices ranging from 0.659 to 0.951. In four patients, carriage–infection dyads were detected within a maximum interval of 48 hours (Table 1). Whole-genome sequencing (WGS) of 12 clonally related isolates identified four sequence types (ST25, ST45, ST307, and ST1161) and corresponding sublineages and clonal groups (Table 2). ST307 and ST1161 are recognized as high-risk clones in international surveillance programs. Resistance gene prediction from WGS data confirmed the widespread presence of carbapenemase genes (*blaNDM-7*, *blaKPC-2*), various ESBLs (*blaCTX-M-2*, *blaCTX-M-15*, *blaSHV-110*, *blaTEM-1*), aminoglycoside resistance determinants (*armA*, *rmtD2*, *aadA*, *aac(3)-IIe*), and quinolone resistance genes (*qnrB*, *oqxA*, *oqxB*) (Table S1). Genes conferring resistance to sulfonamides, tetracyclines, chloramphenicol, and fosfomycin were frequently identified. Genes related to metal (arsenic, mercury, copper) and biocide resistance were also prevalent, potentially enhancing environmental persistence. Virulence profiling showed the widespread presence of iron acquisition systems, such as complete yersiniabactin operons and enterobactin biosynthesis genes, outer membrane protein *ompA*, and the *ecp* pilus operon. However, classical hypervirulence markers (e.g., *rmpA*, *rmpA2*) were absent (10, 28, 29). Among the 12 WGS isolates, Kleborate identified frequent porin alterations consistent with reduced β -lactam permeability (Table S2). *ompK35* was predicted to be disrupted (partial/absent calls ranging from 0-40% of the expected gene length; median 40%) in 10/12 isolates, whereas two isolates (K26 and K28) showed no porin alterations flagged by Kleborate. The *ompK36*GD loop-3 insertion was detected in 8/12 isolates, and one additional isolate (K43.1) displayed a partial *ompK36* call (75%). Overall, 8/12 isolates carried both a disrupted *ompK35* call and *ompK36*GD, suggesting porin-mediated permeability reduction as a common feature in this dataset.

**Figure 4:**
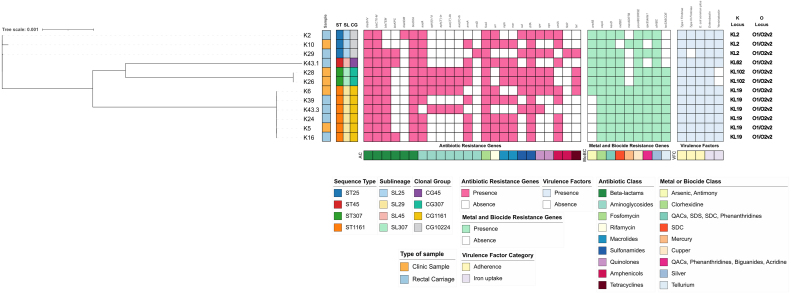
Core genome phylogenetic tree of CR-Kp isolates. Phylogenetic tree and genomic profile of resistance and virulence genes of the CR-Kp strains described in this study (created using the IToL platform).

**Figure 5:**
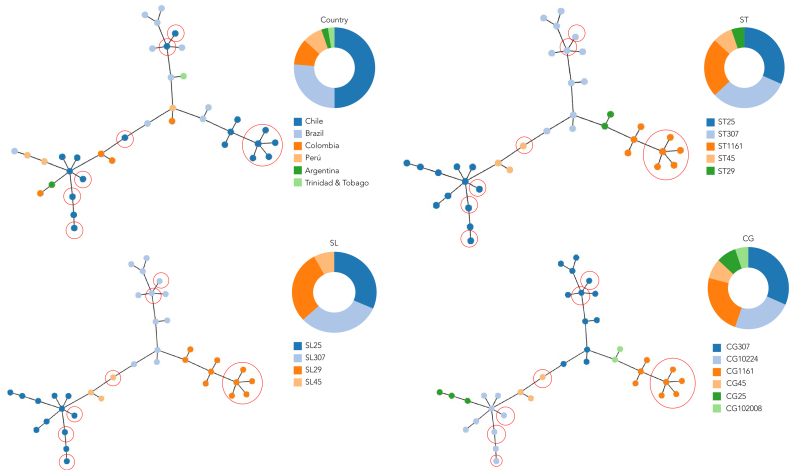
Minimum spanning tree (MST) of South American *K. pneumoniae* strains and the CR-Kp strains described in this study. **Top left**: distribution by **country**; **bottom left**: distribution by **SL** (sublineage); top right: distribution by **ST** (sequence type); **bottom right**: distribution by **CG** (clonal group). Strains from this study are highlighted in red circles. The figure was generated using the PHYLOViZ platform.

Characterization of capsule (K-locus) and O-antigen (O-locus) types revealed the dominance of KL2 and KL19 capsule types and O1/O2v2 variants (Table S3). A phylogenetic analysis based on core-genome alignments showed genetic clustering among the isolates (Figure 4), and comparison with South American strains highlighted links to isolates from Brazil, Colombia, Peru, Argentina, and Trinidad and Tobago (Figure 5). Furthermore, a large-scale phylogenetic tree was generated, including all 579 South American *K. pneumoniae* RefSeq genomes (Figure S4). Clinical and epidemiological data for 26 patients are summarized in Table S4. The cohort had a mean age of 59 years and was predominantly male (54%). Most isolates originated from ICU patients (72%), mainly from rectal surveillance cultures. Common comorbidities included hypertension (46%), diabetes mellitus (19%), and chronic kidney disease (19%). The overall mortality rate was 31%. Statistical analysis showed that *bla*KPC was significantly associated with more extended hospital stays ( p=0.012 ), but no significant associations were observed for age or sex (Table 3). Associations between virulence/resistance genes and clinical variables such as previous invasive procedures were explored. A near-significant trend was found between *bla*TEM carriage and history of surgery or enteroscopy ( p=0.053 and 0.050, respectively) (Table S5). Future studies with larger cohorts are needed to validate these findings.

**Table 1:** Distribution of clonal *Klebsiella pneumoniae* strains by patient, specimen source, isolation date, and originating unit .

**Strain**	**Code**	**Specimen source**	**Patient ID**	**Isolation date**	**Originating Unit**
K19	1	Rectal Swab	PN02	20-09-2021	ICU 2C
K2	1	Endotracheal aspirate	PN02	22-09-2021	ISCU
K13	2	Rectal Swab	PN05	10-01-2022	ICU 2C
K43.3	2	Rectal Swab	PN06	21-02-2022	ICU 2C
K28	3	Oropharyngeal aspirate	PN10	25-11-2021	ICU 2C
K23	3	Oropharyngeal aspirate	PN10	26-11-2021	IMCU
K3	4	Rectal Swab	PN12	11-01-2022	ISCU
K10	4	Oropharyngeal aspirate	PN12	12-01-2022	ISCU
K43.1	5	Rectal Swab	PN06	21-02-2022	ICU 2C
K43.2	5	Rectal Swab	PN06	21-02-2022	ICU 2C
K29	6	Rectal Swab	PN15	04-10-2021	ICU 2C
K30	6	Blood culture	PN01	30-10-2021	ICU 2C
K39	7	Rectal Swab	PN19	26-10-2021	ICU 2C
K8	7	Rectal Swab	PN20	22-09-2021	ICU 2C
K14	8	Rectal Swab	PN21	20-09-2021	ICU 2C
K6	8	Rectal Swab	PN20	25-09-2021	ICU 2C
K26	9	Abdominal collection	PN22	03-11-2021	ICU 2C
K16	9	Rectal Swab	PN22	04-11-2021	ICU 2C
K24	9	Rectal Swab	PN23	25-11-2021	ICU 2C
K5	9	Oropharyngeal aspirate	PN23	26-11-2021	ICU 2C

**Abbreviations:** ICU, Intensive care unit; IMCU, Intermediate medical care unit; ISCU, Intermediate surgical care unit.

**Table 2:** **Typing results based on cgLIN, ST, SL, and CG.** Whole-genome sequencing (WGS) of 12 clonally related isolates identified four sequence types (ST25, ST45, ST307, and ST1161) and corresponding sublineages and clonal groups.

ID	Code	cgLIN	ST	SL	CG
contigsK10	K10	0_0_388_0_1_5_0_0_0_0	ST25	SL25	CG10224
contigsK16	K16	0_0_137_8_2_1_0_0_0_0	ST1161	SL29	CG1161
contigsK2	K2	0_0_388_0_1_5_0_0_0_0	ST25	SL25	CG10224
contigsK24	K24	0_0_137_8_2_1_0_0_0_0	ST1161	SL29	CG1161
contigsK26	K26	0_0_369_0_0_44_0_0_0_0	ST307	SL307	CG307
contigsK28	K28	0_0_369_0_0_44_0_0_0_0	ST307	SL307	CG307
contigsK29	K29	0_0_388_0_1_5_0_0_0_0	ST25	SL25	CG10224
contigsK39	K39	0_0_137_8_2_1_0_0_0_0	ST1161	SL29	CG1161
contigsK431	K43.1	0_0_158_8_6_19_0_0_0_0	ST45	SL45	CG45
contigsK433	K43.3	0_0_137_8_2_1_0_0_0_0	ST1161	SL29	CG1161
contigsK5	K5	0_0_137_8_2_1_0_0_0_0	ST1161	SL29	CG1161
contigsK6	K6	0_0_137_8_2_1_0_0_0_0	ST1161	SL29	CG1161

**Abbreviations: cgLIN**, Core Genome Life Identification Number; **ST**, Sequence Type; **SL**, Sublineage; **CG**, Clonal Group.

**Table 3:** Summary of results from Student’s *t*-tests and Mann–Whitney *U* tests. Statistical significance level: *p*-value < 0.05. Compiled from the CSV file generated in RStudio, version 2024.09.0+375.

Factor	*blaKPC* ( − )	*blaKPC* ( + )	*p-value*
Age (SD)	61.67 (19.35)	57.39 (15.04)	0.54
Gender	4 (67%)	22 (58%)	1.00
Length of hospital stay (Median, IQR)	34.00 (26.00, 59.00)	93.00 (60.00, 164.00)	**0.012**

**Abbreviations:** SD, Standard deviation; IQR, Interquartile range. Associations between virulence/resistance genes and clinical variables such as previous invasive procedures were explored. A near-significant trend was found between *bla*TEM carriage and history of surgery or enteroscopy ( p=0.053 and 0.050, respectively) (Table S4). However, the limited sample size (n=44 isolates) warrants caution in interpreting these trends. Future studies with larger cohorts are needed to validate these findings.

## DISCUSSION

This study reveals a complex antimicrobial-resistance landscape in *Klebsiella pneumoniae*. Phenotypic testing showed high resistance to β -lactams, fluoroquinolones, and aminoglycosides, consistent with MDR/XDR phenotypes and the high prevalence of ESBL and carbapenemase genes detected by PCR. These patterns severely limit therapeutic options. The predominance of *blaKPC* mirrors regional and global trends identifying KPC enzymes as the main carbapenem-resistance drivers in South America 34, 35 and aligns with Chilean reports since 2014 33. Lower frequencies of *blaNDM* and *blaVIM* (4% and 9%) suggest diversification of mechanisms, likely favored by plasmid mobility and selective pressure from carbapenem use in critical care 36. The high XDR proportion underscores the urgency for new therapies and sustained surveillance. Detection of *blaOXA-1and* *blaOXA-10* raises questions about their contribution: unlike OXA-48, these enzymes lack intrinsic carbapenemase activity 18, but, combined with porin loss or mutations (e.g., OmpK35/OmpK36), may further reduce carbapenem susceptibility. Consistent with this, Kleborate identified porin alterations in most WGS isolates (e.g., *ompK35* disruption in 10/12 and *ompK36*GD in 8/12), suggesting that decreased outer-membrane permeability may contribute, alongside β -lactamases, to reduced carbapenem susceptibility. We also observed discordance in two isolates that were carbapenemase-positive by routine phenotypic testing, yet negative for carbapenemase genes in our PCR panel. Importantly, we did not pursue additional investigations (e.g., WGS, expanded carbapenemase panels, porin/efflux assessment, or plasmid analysis) to determine the underlying mechanism in these isolates. Although plasmid reconstruction was beyond the scope of this study due to limitations of short-read sequencing, the distribution of resistance genes strongly suggests plasmid-mediated dissemination. Future long-read sequencing approaches will be required to resolve the structure and transmission dynamics of mobile genetic elements. Therefore, the basis of carbapenem resistance in this small subset remains undetermined and is acknowledged as a limitation of the study.

Virulence-gene analysis identified iron-acquisition systems (yersiniabactin *ytsB*, enterobactin *entB*, aerobactin *iutA*) and a high prevalence of the adhesin *mrkD*, features that may promote persistence in hospitals and hosts. Although typical of classical *K. pneumoniae* (cKp) 29, their coexistence with carbapenem-resistance genes is concerning, suggesting resistance–virulence convergence. Notably, classical hypervirulence markers (*rmpA*, *rmpA2*, *magA*, *peg*-*344*) were absent by PCR and WGS, indicating the *hvKp* phenotype is not predominant. Detection of the *K2* capsular serotype—often linked to hypervirulence—may enhance biofilm formation and resistance to host defenses 28. These findings support the need for complete genome sequencing and advanced bioinformatics to refine pathogenic-potential assessments 30. Epidemiologically, PFGE indicated multiple clones, and WGS identified four sequence types (ST25, ST45, ST307, ST1161), reflecting high diversity. Carbapenemase genes were widespread across clones, indicating no clonal restriction and intense horizontal gene transfer 15, 16. Genetically similar isolates in different patients suggest possible patient-to-patient transmission, though formal outbreak declarations require strict criteria. In four cases, carriage-infection dyads occurred <48 h apart. In three, rectal colonization preceded the clinical isolate; in patient PN22, the sequence reversed, implying initially undetected carriage or emergence after abdominal infection under antibiotic pressure or surgical manipulation. These patterns illustrate bidirectional dynamics between colonization and sterile-site infection. Thus, rectal surveillance is essential; the PN22 timeline shows that an initial negative screen does not preclude later infection. The concentration of isolates in ICU 2C, together with these dyads, supports continuous surveillance as an early-warning system and a basis for downstream genotyping to confirm or exclude clonal relatedness. Historically, CG258/ST258 dominated carbapenemase dissemination 37, but recent reports show the rise of non-CG258 CR-Kp, notably ST307, a high-risk clone detected here 38. Initially associated with *bla*CTX-M-15, ST307 has increasingly acquired carbapenemases and multidrug resistance and may supplant ST258 globally 20. Other STs merit attention: ST25 has been implicated in invasive infections in Argentina and Ecuador 20, 39 and, recently, in Chile 33, often with K2, suggesting resistance–virulence convergence; ST45, an emerging high-risk clone, has caused outbreaks in Europe and Latin America involving carbapenemase and non-carbapenemase producers 40–43; ST1161, mainly reported in Chile, represents a local lineage with carbapenemase production and prolonged hospital persistence 44, 45. A minimum-spanning-tree analysis indicates that Chilean CR-Kp participates in a broader transnational dynamic: genetic proximity to strains from Brazil, Colombia, Peru, Argentina, and Trinidad and Tobago suggests globalization facilitates spread across South America. These data argue for active surveillance in patients with international healthcare exposure and regional coordination in genomic surveillance and antimicrobial stewardship. Despite sample-size limitations, statistical analyses yielded relevant signals. The strongest association linked *blaKPC* carriage to longer hospital stays, consistent with prior work relating KPC producers to delayed effective therapy, more complications, and prolonged hospitalization 46–48. No significant associations emerged for other genes (e.g., *k2*, *mrkD*, *ytsB*, *blaNDM*, *blaVIM*) with age, comorbidities, or device use. While the cohort size limits statistical power, the absence of associations for several determinants may also reflect low feature variability, as multiple genes were nearly ubiquitous across isolates (e.g., *blaCTX-M, blaSHV, entB, iutA*), reducing contrast between comparison groups. Accordingly, negative association results should be interpreted cautiously.

Beyond carbapenem resistance, genomic analyses revealed a wide repertoire of resistance determinants affecting aminoglycosides, quinolones, sulfonamides, and heavy metals, suggesting multidimensional selective pressures within hospital environments. These accessory resistance traits may enhance persistence and ecological fitness independently of carbapenemase carriage.

In conclusion, although only the *blaKPC*-hospitalization link reached statistical significance, observed trends highlight the complexity of CR-Kp infections and the interplay between bacterial genetics, clinical practices, and resistance evolution. Expanding cohorts and longitudinal monitoring will better capture dissemination dynamics and outcomes. Our findings illuminate the molecular epidemiology of carbapenem-resistant *K. pneumoniae* in Chile and reinforce the need for ongoing genomic surveillance and integrated infection-control strategies. This molecular characterization clarifies local resistance mechanisms and virulence profiles, informing management of critically ill patients. Integrating molecular epidemiology into clinical practice can guide treatment and strengthen prevention measures against the growing challenge of antimicrobial resistance.

## MATERIAL AND METHODS

### Bacterial isolates

Forty-five carbapenem-resistant *Klebsiella pneumoniae* isolates were obtained from 26 patients hospitalized in critical care units at the Clinical Hospital of the University of Chile (HCUCH) between September 2021 and May 2022. Isolates were recovered from clinical samples and rectal colonization surveillance swabs, including one environmental isolate. Species identification was performed using VITEK MS (bioMérieux, France) with 99.9% confidence. Carbapenemase production was confirmed using the CarbaNP test and immunochromatographic assay (O.K.N.V.I RESIST-5, Coris BioConcept).

### Antibiotic susceptibility testing

Antibiotic susceptibility was assessed using the Kirby-Bauer disk diffusion method on Mueller-Hinton agar. Antibiotics tested are listed in Table S6. Interpretation of results (Susceptible [S], Intermediate [I], Dose-dependent susceptibility [SDD], Resistant [R]) followed CLSI M100 guidelines, 33rd edition 49. ESBL detection was based on a ≥ 5 mm increase in inhibition zones with clavulanic acid combinations. Strains were classified as MDR, XDR, or PDR following PAHO definitions 50.

### Molecular detection of resistance and virulence genes

Strains were stored at − 80 ${}^{\circ }$ C, streaked on MacConkey agar, and a single colony was grown overnight at 37 ${}^{\circ }$ C in 3 mL LB Lennox (120 rpm). Genomic DNA was extracted with the Wizard® Genomic DNA Purification Kit (Promega, USA) and resuspended to 100 μ L. Concentration/purity was measured on a BioTek Synergy HT (Gen5 v2.09; Agilent, USA); an acceptable A260/280 was 1.8–2.0.

Carbapenemase genes were assessed by two multiplex PCRs: *blaKPC*, *blaNDM*, *blaOXA*-*48* (set 1) and *blaIMP*, *blaVIM* (set 2); ESBL genes (*blaTEM*, *blaSHV*, *blaCTX*-*M*) were amplified by multiplex PCR (primers in Tables S7–S8). Each 20 μ L reaction contained 0.8 μ L template, 0.4 μ L each primer, 4 μ L 5 × GoTaq® buffer, and 0.1 μ L GoTaq® DNA polymerase (Promega). Cycling: 95 ${}^{\circ }$ C 2 min; 35 cycles of 95 ${}^{\circ }$ C 1 min, 56 ${}^{\circ }$ C 1 min, 72 ${}^{\circ }$ C 1 min; final 72 ${}^{\circ }$ C 5 min.

Virulence genes were detected by multiplex PCR targeting *ytsB*, *entB*, *iutA*, *kfu*, *allS*, *mrkD*, *rmpA*, *k2*, and *magA* (primers in Table S9). Amplicons were resolved on 2% agarose gels stained with ethidium bromide.

### Clonality and genomic characterization

Pulsed-field gel electrophoresis (PFGE) was used to assess clonal relatedness following XbaI digestion. DNA plugs were prepared from bacterial cultures embedded in agarose, lysed, digested, and separated using a CHEF-DR III system (Bio-Rad). Banding patterns were analyzed with GelCompar II software using the Dice coefficient and UPGMA clustering with 3% tolerance.

### Whole genome sequencing and analysis

MicrobesNG (Birmingham, UK) sequenced genomic DNA from clonally related isolates on an Illumina platform. Quality control was performed using FastQC v0.11.9 51. Genomes were assembled de novo with SPAdes v3.13.1 52 and evaluated using QUAST v5.2.0 53. Annotation was done with Prokka v1.14.6 54. Contigs shorter than 200 nt were removed, and sequences were deposited at GenBank under Bioproject PRJNA1315165.

### Resistance and virulence gene prediction

Resistance genes were predicted using ABRicate v1.0.1 55 with the NCBI and ResFinder databases 56, 57. The Kleborate 42 and BACMET 58 databases were used for additional predictions of resistance to biocides and metals. Virulence factors were identified using Kleborate and VFDB 59.

### Sequence typing and clonal assignment

MLST was performed with the MLST v2.23.0 tool 60 using PubMLST databases 61. cgLIN codes were assigned following hierarchical clustering principles based on cgMLST profiles 62, utilizing chewBBACA v3.3.10 63 for allele calling and assignment.

### Core genome alignment and phylogenetic analysis

Core genome alignment was generated using Roary v.3.13.0 64, applying the default core-genome definition (genes present in ≥ 95% of isolates). The resulting core-genome alignment (N core genes; alignment length: X bp) was used to infer a phylogeny with FastTree 65, a maximum-likelihood-based approach, under the GTR+ Γ substitution model (GTR with discrete gamma rate heterogeneity). The gamma option was enabled to account for among-site rate variation and to improve branch-length estimation. The double-precision build (FastTreeDbl) was used to enhance numerical stability in likelihood calculations. Trees were visualized using the Interactive Tree of Life (iTOL) 66.

### Comparative genomics and Minimum Spanning Tree (MST)

South American *K. pneumoniae* genomes ( n=579 ) were downloaded from RefSeq and filtered for quality based on Hennart et al. (2022) criteria. The list of RefSeq assembly accessions included in the regional phylogenetic analyses is provided in Table S10. Closely related genomes ( ≥ 80% similarity) were selected for MST construction using PHYLOViZ Online 67 with the goeBURST algorithm 68.

### Clinical data collection and statistical analysis

Clinical data were collected under ethics approval (Resolution No. 56, HCUCH) and included demographics, hospitalization details, comorbidities, invasive procedures, and outcomes. Statistical analyses were conducted using RStudio (v2024.09.0+375). Normality was assessed by the Shapiro-Wilk test. Depending on data distribution, the Student’s t-test or the Mann-Whitney U test was applied for continuous variables. Fisher’s exact test was used for categorical variables. Associations with p < 0.05 were considered statistically significant.

## ETHICS

The clinical data of the patients from whom the studied strains were isolated were collected from patient records, following prior approval by the Scientific Ethics Committee of the Hospital Clínico Universidad de Chile through Resolution No. 56, 04-10-2023. The request was made through a letter of amendment and the submission of the “Request for access to clinical records” form, following the protocols established by the Committee.

## AUTHORS CONTRIBUTIONS

Conceptualization and experimental design: FS and RV; Data acquisition: GA, AA, CA, BB, and FS; Data analysis and interpretation: GA, FS, RB, AJ, and RV; Writing—original draft: RV; Review: GA, FS, and RV. Final Edition: GA, FS, AJ, and RV. All authors have read and agreed to the published version of the manuscript.

## SUPPLEMENTAL MATERIAL

All supplemental data for this article are available online at http://microbialcell.com/researcharticles/2026a-araya-microbial-cell/.

.

## FUNDING

This work was funded by Fondo Nacional de Desarrollo Científico y Tecnológico (FONDECYT) grant number 1211647, awarded to Vidal RM and “Líneas de Apoyo a la Investigación Financiadas por el ICBM (2026)” Instituto de Ciencias Biomédicas (ICBM), Facultad de Medicina, Universidad de Chile.

## CONFLICT OF INTEREST

The authors declare that they have no conflict of interest.
