# Supplementary material for: Genomic epidemiology of carbapenemase-producing Klebsiella pneumoniae circulating in a Chilean tertiary-care hospital (2021–2022): Molecular characterization, resistance-virulence convergence, and clinical associations
Source: Microb Cell. 2026 May 21;13:186–97. doi: 10.15698/mic2026.05.876 (PMC13270816; doi:10.15698/mic2026.05.876)
Supplement: Supplementary file 1 — . [file mic-13-186-s01.pdf]

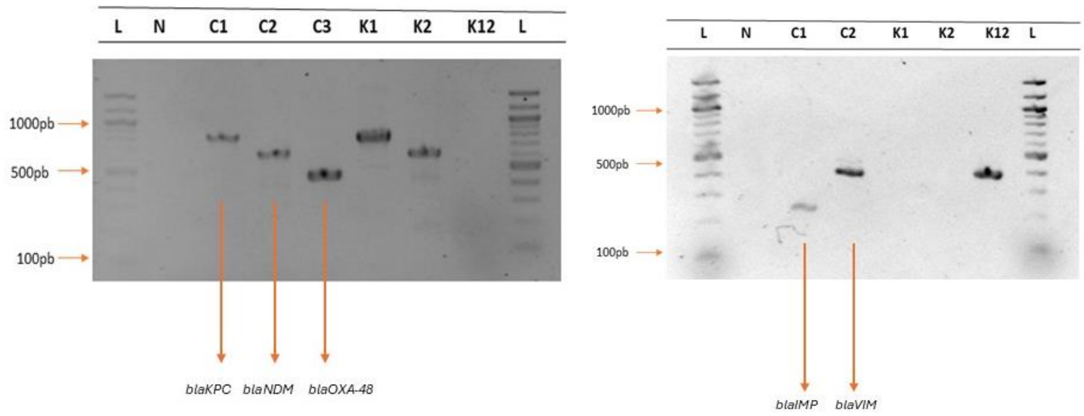

Figure S1 (S1A and S1B): Multiplex PCR assay for the detection of carbapenemase genes. On the left (S1A), assay for the detection of ***blaKPC (*798 bp), *blaNDM* (621 bp),** and ***blaOXA-48* (438 bp)** genes. **L**: 100 bp ladder; **N**: Negative control; **C1**: *K. pneumoniae* BAA-1705 *blaKPC* (+); **C2**: *K. pneumoniae* UCO-361 *blaNDM* (+); **C3**: *K. pneumoniae* BAA-2524 *blaOXA-48* (+). On the right (S1B), assay for the detection of the ***blaIMP* (232 bp)** and ***blaVIM* (390 bp)** genes. **L**: 100 bp ladder; **N**: Negative control; **C1**: *S. marcescens* UCO-143 *blaIMP* (+); **C2**: *P. aeruginosa blaVIM* (+).

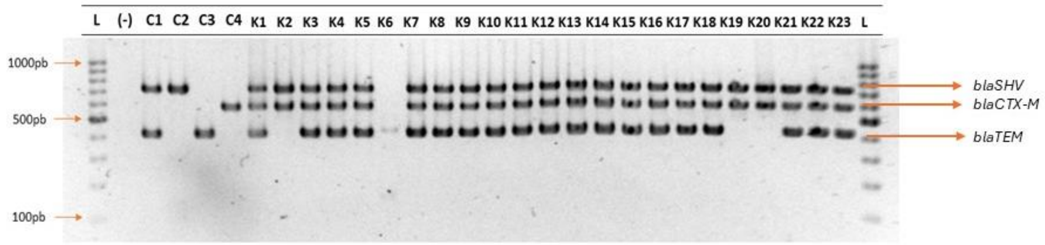

Figure S2: Multiplex PCR assay for the detection of *blaTEM* (422 bp), *blaSHV* (739 bp), and *blaCTX-M* (593 bp) genes. L: 100 bp ladder; **(–)**: Negative control; **C1**: *K. pneumoniae* BAA-1705 *blaTEM* (+), *blaSHV* (+); **C2**: *K. pneumoniae* ATCC 700603 *blaSHV* (+); **C3**: *E. coli* ATCC 35218 *blaTEM* (+); **C4**: *E. coli* UCH-235 *blaCTX-M* (+).

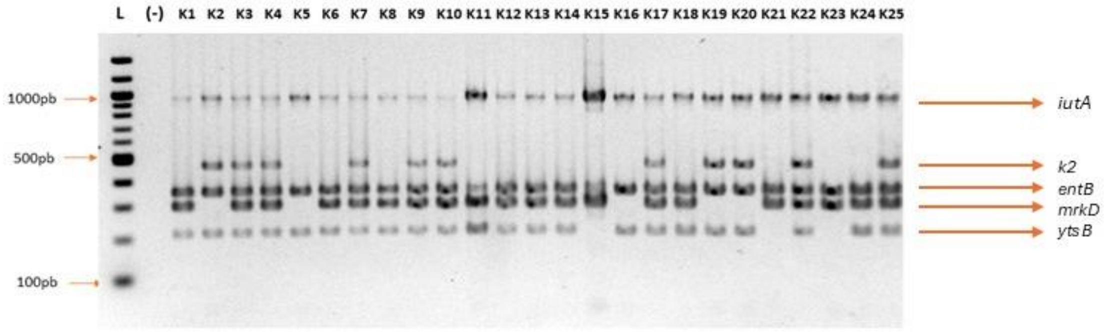

Figure S3. Multiplex PCR for virulence genes. Multiplex PCR assay for the detection of *ytsB* (242 bp), *entB* (400 bp), *iutA* (920 bp), *kfu* (638 bp), *allS* (764 bp), *mrkD* (340 bp), *rmpA* (461 bp), *k2* (531 bp), and ***magA* (1283 bp) genes. L**: 100 bp ladder; **(–)**: Negative control.

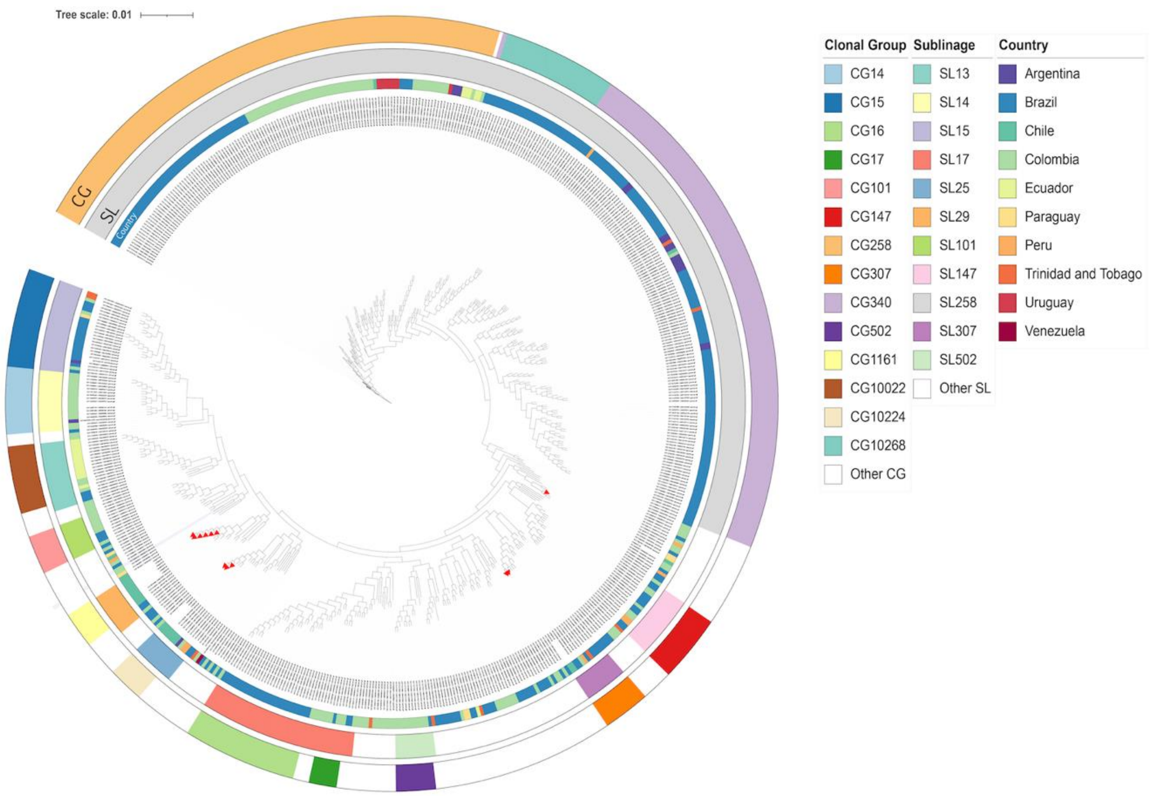

Figure S4. Phylogenetic tree of South American *K. pneumoniae* strains and the CR-Kp strains sequenced and described in this study. Red arrows indicate strains from this study. The figure was created using the IToL platform.

Table S1. Resistance genes identified in CR-Kp isolates. Compiled from the **TSV** file generated by the **ABRicate** and **Kleborate** platforms.

GENE	%COVERAGE	%IDENTITY	PRODUCT	STRAIN
blaSHV-110	100	100	class A beta-lactamase SHV-110	K2
fosA6	100	99	fosfomycin resistance glutathione transferase FosA6	
qnrB19	100	100	quinolone resistance pentapeptide repeat protein QnrB19	
oqxB17	100	99	multidrug efflux RND transporter permease subunit OqxB17	
oqxA7	100	100	multidrug efflux RND transporter periplasmic adaptor subunit OqxA7	
blaNDM-7	100	100	subclass B1 metallo-beta-lactamase NDM-7	
ble-MBL	100	100	bleomycin binding protein Ble-MBL	
blaCTX-M-2	100	100	class A extended-spectrum beta-lactamase CTX-M-2	
sul1	100	100	sulfonamide-resistant dihydropteroate synthase Sul1	
aadA1	100	100	ANT(3")-Ia family aminoglycoside nucleotidyltransferase AadA1	
blaOXA-10	100	100	oxacillin-hydrolyzing class D beta-lactamase OXA-10	
cmlA5	100	100	chloramphenicol efflux MFS transporter CmlA5	
arr-2	100	100	NAD(+)--rifampin ADP-ribosyltransferase Arr-2	
dfrA14	100	100	trimethoprim-resistant dihydrofolate reductase DfrA14	
blaCTX-M-15	100	100	class A extended-spectrum beta-lactamase CTX-M-15	
blaCTX-M-3	100.00	100.00	class A extended-spectrum beta-lactamase CTX-M-3	K5
blaTEM-1	100.00	100.00	class A broad-spectrum beta-lactamase TEM-1	
blaSHV-187	100.00	100.00	class A beta-lactamase SHV-187	

armA	100.00	100.00	ArmA family 16S rRNA (guanine(1405)-N(7))-methyltransferase	
msr(E)	100.00	100.00	ABC-F type ribosomal protection protein Msr(E)	
mph(E)	100.00	100.00	Mph(E) family macrolide 2'-phosphotransferase	
aadA1	100.00	99.87	ANT(3")-Ia family aminoglycoside nucleotidyltransferase AadA1	
blaOXA-10	100.00	100.00	oxacillin-hydrolyzing class D beta-lactamase OXA-10	
cmlA5	100.00	100.00	chloramphenicol efflux MFS transporter CmlA5	
arr-2	100.00	100.00	NAD(+)--rifampin ADP-ribosyltransferase Arr-2	
blaCTX-M-2	100.00	100.00	class A extended-spectrum beta-lactamase CTX-M-2	
aadA2	100.00	100.00	ANT(3")-Ia family aminoglycoside nucleotidyltransferase AadA2	
dfrA12	100.00	100.00	trimethoprim-resistant dihydrofolate reductase DfrA12	
fosA6	100.00	99.29	fosfomycin resistance glutathione transferase FosA6	
blaCTX-M-3	100.00	100.00	class A extended-spectrum beta-lactamase CTX-M-3	K6
blaTEM-1	100.00	99.88	class A broad-spectrum beta-lactamase TEM-1	
blaKPC-2	100.00	100.00	carbapenem-hydrolyzing class A beta-lactamase KPC-2	
mph(A)	100.00	99.67	Mph(A) family macrolide 2'-phosphotransferase	
blaSHV-187	100.00	100.00	class A beta-lactamase SHV-187	
mph(E)	100.00	100.00	Mph(E) family macrolide 2'-phosphotransferase	
msr(E)	100.00	100.00	ABC-F type ribosomal protection protein Msr(E)	
armA	100.00	100.00	ArmA family 16S rRNA (guanine(1405)-N(7))-methyltransferase	
aadA1	100.00	99.87	ANT(3")-Ia family aminoglycoside nucleotidyltransferase AadA1	
blaOXA-10	100.00	100.00	oxacillin-hydrolyzing class D beta-lactamase OXA-10	
cmlA5	100.00	100.00	chloramphenicol efflux MFS transporter CmlA5	

arr-2	100.00	100.00	NAD(+)--rifampin ADP-ribosyltransferase Arr-2	
blaCTX-M-2	100.00	100.00	class A extended-spectrum beta-lactamase CTX-M-2	
aadA2	100.00	100.00	ANT(3")-Ia family aminoglycoside nucleotidyltransferase AadA2	
dfrA12	100.00	100.00	trimethoprim-resistant dihydrofolate reductase DfrA12	
fosA6	100.00	99.29	fosfomycin resistance glutathione transferase FosA6	
blaSHV-110	100.00	99.77	class A beta-lactamase SHV-110	K10
dfrA14	100.00	100.00	trimethoprim-resistant dihydrofolate reductase DfrA14	
blaCTX-M-30	100.00	99.77	class A extended-spectrum beta-lactamase CTX-M-30	
qnrB19	100.00	100.00	quinolone resistance pentapeptide repeat protein QnrB19	
blaOXA-9	100.00	100.00	oxacillin-hydrolyzing class D beta-lactamase OXA-9	
aadA1	100.00	100.00	ANT(3")-Ia family aminoglycoside nucleotidyltransferase AadA1	
blaKPC-2	100.00	100.00	carbapenem-hydrolyzing class A beta-lactamase KPC-2	
oqxB17	100.00	99.24	multidrug efflux RND transporter permease subunit OqxB17	
oqxA7	100.00	99.92	multidrug efflux RND transporter periplasmic adaptor subunit OqxA7	
rmtD2	100.00	100.00	16S rRNA (guanine(1405)-N(7))-methyltransferase RmtD2	
floR	99.84	85.24	chloramphenicol/florfenicol efflux MFS transporter FloR	
tet(G)	100.00	94.13	tetracycline efflux MFS transporter Tet(G)	
fosA6	100.00	99.05	fosfomycin resistance glutathione transferase FosA6	
blaOXA-101	100.00	100.00	OXA-10 family class D beta-lactamase OXA-101	
blaTEM-150	100.00	99.88	class A beta-lactamase TEM-150	
sul1	100.00	100.00	sulfonamide-resistant dihydropteroate synthase Sul1	

blaCTX-M-3	100.00	100.00	class A extended-spectrum beta-lactamase CTX-M-3	K16
blaCTX-M-2	100.00	100.00	class A extended-spectrum beta-lactamase CTX-M-2	
blaTEM-1	100.00	100.00	class A broad-spectrum beta-lactamase TEM-1	
blaSHV-187	100.00	100.00	class A beta-lactamase SHV-187	
armA	100.00	100.00	ArmA family 16S rRNA (guanine(1405)-N(7))-methyltransferase	
msr(E)	100.00	100.00	ABC-F type ribosomal protection protein Msr(E)	
mph(E)	100.00	100.00	Mph(E) family macrolide 2'-phosphotransferase	
aadA1	100.00	99.87	ANT(3")-Ia family aminoglycoside nucleotidyltransferase AadA1	
blaOXA-10	100.00	100.00	oxacillin-hydrolyzing class D beta-lactamase OXA-10	
cmlA5	100.00	100.00	chloramphenicol efflux MFS transporter CmlA5	
arr-2	100.00	100.00	NAD(+)--rifampin ADP-ribosyltransferase Arr-2	
dfrA12	100.00	100.00	trimethoprim-resistant dihydrofolate reductase DfrA12	
aadA2	100.00	100.00	ANT(3")-Ia family aminoglycoside nucleotidyltransferase AadA2	
fosA6	100.00	99.29	fosfomycin resistance glutathione transferase FosA6	
blaSHV-187	100.00	100.00	class A beta-lactamase SHV-187	K24
blaKPC-2	100.00	100.00	carbapenem-hydrolyzing class A beta-lactamase KPC-2	
armA	100.00	100.00	ArmA family 16S rRNA (guanine(1405)-N(7))-methyltransferase	
msr(E)	100.00	100.00	ABC-F type ribosomal protection protein Msr(E)	
mph(E)	100.00	100.00	Mph(E) family macrolide 2'-phosphotransferase	
blaCTX-M-2	100.00	100.00	class A extended-spectrum beta-lactamase CTX-M-2	
blaCTX-M-3	100.00	100.00	class A extended-spectrum beta-lactamase CTX-M-3	

aadA1	100.00	99.87	ANT(3")-Ia family aminoglycoside nucleotidyltransferase AadA1	
blaOXA-10	100.00	100.00	oxacillin-hydrolyzing class D beta-lactamase OXA-10	
cmlA5	100.00	100.00	chloramphenicol efflux MFS transporter CmlA5	
arr-2	100.00	100.00	NAD(+)--rifampin ADP-ribosyltransferase Arr-2	
aadA2	100.00	100.00	ANT(3")-Ia family aminoglycoside nucleotidyltransferase AadA2	
dfrA12	100.00	100.00	trimethoprim-resistant dihydrofolate reductase DfrA12	
blaOXA-9	100.00	99.88	oxacillin-hydrolyzing class D beta-lactamase OXA-9	
blaTEM-1	100.00	99.77	class A broad-spectrum beta-lactamase TEM-1	
fosA6	100.00	99.29	fosfomycin resistance glutathione transferase FosA6	
blaSHV-106	100.00	99.88	class A extended-spectrum beta-lactamase SHV-106	K26
mph(E)	100.00	100.00	Mph(E) family macrolide 2'-phosphotransferase	
msr(E)	100.00	100.00	ABC-F type ribosomal protection protein Msr(E)	
armA	100.00	100.00	ArmA family 16S rRNA (guanine(1405)-N(7))-methyltransferase	
sul1	100.00	100.00	sulfonamide-resistant dihydropteroate synthase Sul1	
aadA2	100.00	100.00	ANT(3")-Ia family aminoglycoside nucleotidyltransferase AadA2	
dfrA12	100.00	100.00	trimethoprim-resistant dihydrofolate reductase DfrA12	
tet(A)	100.00	100.00	tetracycline efflux MFS transporter Tet(A)	
qnrB1	100.00	100.00	quinolone resistance pentapeptide repeat protein QnrB1	
dfrA14	100.00	100.00	trimethoprim-resistant dihydrofolate reductase DfrA14	
aph(6)-Id	100.00	100.00	aminoglycoside O-phosphotransferase APH(6)-Id	
aph(3")-Ib	100.00	100.00	aminoglycoside O-phosphotransferase APH(3")-Ib	
sul2	100.00	100.00	sulfonamide-resistant dihydropteroate synthase Sul2	
aac(3)-IIe	100.00	99.77	aminoglycoside N-acetyltransferase AAC(3)-IIe	

blaOXA-1	100.00	100.00	oxacillin-hydrolyzing class D beta-lactamase OXA-1	
aac(6')-Ib-D181Y	100.00	99.82	AAC(6')-Ib family aminoglycoside 6'-N-acetyltransferase	
oqxB19	100.00	99.40	multidrug efflux RND transporter permease subunit OqxB19	
oqxA5	100.00	99.41	multidrug efflux RND transporter periplasmic adaptor subunit OqxA5	
blaCTX-M-3	100.00	100.00	class A extended-spectrum beta-lactamase CTX-M-3	
blaTEM-1	100.00	100.00	class A broad-spectrum beta-lactamase TEM-1	
fosA6	100.00	99.76	fosfomycin resistance glutathione transferase FosA6	
blaSHV-106	100.00	99.88	class A extended-spectrum beta-lactamase SHV-106	K28
dfrA12	100.00	100.00	trimethoprim-resistant dihydrofolate reductase DfrA12	
aadA2	100.00	100.00	ANT(3")-Ia family aminoglycoside nucleotidyltransferase AadA2	
sul1	100.00	100.00	sulfonamide-resistant dihydropteroate synthase Sul1	
armA	100.00	100.00	ArmA family 16S rRNA (guanine(1405)-N(7))-methyltransferase	
msr(E)	100.00	100.00	ABC-F type ribosomal protection protein Msr(E)	
mph(E)	100.00	100.00	Mph(E) family macrolide 2'-phosphotransferase	
tet(A)	100.00	100.00	tetracycline efflux MFS transporter Tet(A)	
qnrB1	100.00	100.00	quinolone resistance pentapeptide repeat protein QnrB1	
dfrA14	100.00	100.00	trimethoprim-resistant dihydrofolate reductase DfrA14	
sul2	100.00	100.00	sulfonamide-resistant dihydropteroate synthase Sul2	
aph(3")-Ib	100.00	100.00	aminoglycoside O-phosphotransferase APH(3")-Ib	
aph(6)-Id	100.00	100.00	aminoglycoside O-phosphotransferase APH(6)-Id	
aac(3)-IIe	100.00	99.77	aminoglycoside N-acetyltransferase AAC(3)-IIe	

aac(6')-Ib-D181Y	100.00	99.82	AAC(6')-Ib family aminoglycoside 6'-N-acetyltransferase	
blaOXA-1	100.00	100.00	oxacillin-hydrolyzing class D beta-lactamase OXA-1	
blaCTX-M-3	100.00	100.00	class A extended-spectrum beta-lactamase CTX-M-3	
oqxA5	100.00	99.41	multidrug efflux RND transporter periplasmic adaptor subunit OqxA5	
oqxB19	100.00	99.40	multidrug efflux RND transporter permease subunit OqxB19	
blaTEM-1	100.00	100.00	class A broad-spectrum beta-lactamase TEM-1	
fosA6	100.00	99.76	fosfomycin resistance glutathione transferase FosA6	
blaSHV-110	100.00	99.77	class A beta-lactamase SHV-110	K29
fosA6	100.00	99.05	fosfomycin resistance glutathione transferase FosA6	
dfrA14	100.00	100.00	trimethoprim-resistant dihydrofolate reductase DfrA14	
oqxA7	100.00	99.92	multidrug efflux RND transporter periplasmic adaptor subunit OqxA7	
oqxB17	100.00	99.24	multidrug efflux RND transporter permease subunit OqxB17	
blaCTX-M-2	100.00	100.00	class A extended-spectrum beta-lactamase CTX-M-2	
sul1	100.00	100.00	sulfonamide-resistant dihydropteroate synthase Sul1	
aadA1	100.00	99.87	ANT(3")-Ia family aminoglycoside nucleotidyltransferase AadA1	
blaOXA-10	100.00	100.00	oxacillin-hydrolyzing class D beta-lactamase OXA-10	
cmlA5	100.00	100.00	chloramphenicol efflux MFS transporter CmlA5	
arr-2	100.00	100.00	NAD(+)--rifampin ADP-ribosyltransferase Arr-2	
qnrB19	100.00	100.00	quinolone resistance pentapeptide repeat protein QnrB19	
tet(A)	100.00	100.00	tetracycline efflux MFS transporter Tet(A)	
aph(3")-Ib	95.17	100.00	aminoglycoside O-phosphotransferase APH(3")-Ib	

aph(6)-Id	100.00	100.00	aminoglycoside O-phosphotransferase APH(6)-Id	
blaTEM-1	100.00	100.00	class A broad-spectrum beta-lactamase TEM-1	
qnrB1	100.00	100.00	quinolone resistance pentapeptide repeat protein QnrB1	
aac(3)-IIe	100.00	99.77	aminoglycoside N-acetyltransferase AAC(3)-IIe	
blaOXA-1	100.00	100.00	oxacillin-hydrolyzing class D beta-lactamase OXA-1	
aac(6')-Ib-D181Y	100.00	99.82	AAC(6')-Ib family aminoglycoside 6'-N-acetyltransferase	
sul2	100.00	100.00	sulfonamide-resistant dihydropteroate synthase Sul2	
blaCTX-M-3	100.00	100.00	class A extended-spectrum beta-lactamase CTX-M-3	K39
blaTEM-1	100.00	100.00	class A broad-spectrum beta-lactamase TEM-1	
blaSHV-187	100.00	100.00	class A beta-lactamase SHV-187	
mph(E)	100.00	100.00	Mph(E) family macrolide 2'-phosphotransferase	
msr(E)	100.00	100.00	ABC-F type ribosomal protection protein Msr(E)	
armA	100.00	100.00	ArmA family 16S rRNA (guanine(1405)-N(7))-methyltransferase	
arr-2	100.00	100.00	NAD(+)--rifampin ADP-ribosyltransferase Arr-2	
cmlA5	100.00	100.00	chloramphenicol efflux MFS transporter CmlA5	
blaOXA-10	100.00	100.00	oxacillin-hydrolyzing class D beta-lactamase OXA-10	
aadA1	100.00	99.87	ANT(3")-Ia family aminoglycoside nucleotidyltransferase AadA1	
blaCTX-M-2	100.00	100.00	class A extended-spectrum beta-lactamase CTX-M-2	
aadA2	100.00	100.00	ANT(3")-Ia family aminoglycoside nucleotidyltransferase AadA2	
dfrA12	100.00	100.00	trimethoprim-resistant dihydrofolate reductase DfrA12	

fosA6	100.00	99.29	fosfomycin resistance glutathione transferase FosA6	
blaSHV-145	100.00	99.88	class A beta-lactamase SHV-145	K43.1
dfrA14	100.00	100.00	trimethoprim-resistant dihydrofolate reductase DfrA14	
aac(3)-IIe	100.00	99.77	aminoglycoside N-acetyltransferase AAC(3)-IIe	
qnrB1	100.00	100.00	quinolone resistance pentapeptide repeat protein QnrB1	
sul2	100.00	100.00	sulfonamide-resistant dihydropteroate synthase Sul2	
aph(3'')-Ib	100.00	100.00	aminoglycoside O-phosphotransferase APH(3'')-Ib	
aph(6)-Id	100.00	100.00	aminoglycoside O-phosphotransferase APH(6)-Id	
blaTEM-1	100.00	100.00	class A broad-spectrum beta-lactamase TEM-1	
blaOXA-1	100.00	100.00	oxacillin-hydrolyzing class D beta-lactamase OXA-1	
aac(6')-Ib-D181Y	100.00	99.82	AAC(6')-Ib family aminoglycoside 6'-N-acetyltransferase	
blaCTX-M-15	100.00	100.00	class A extended-spectrum beta-lactamase CTX-M-15	
oqxA11	100.00	99.66	multidrug efflux RND transporter periplasmic adaptor subunit OqxA11	
oqxB20	100.00	99.11	multidrug efflux RND transporter permease subunit OqxB20	
fosA_gen	100.00	100.00	FosA family fosfomycin resistance glutathione transferase	
blaCTX-M-3	100.00	100.00	class A extended-spectrum beta-lactamase CTX-M-3	K43.3
blaTEM-1	100.00	100.00	class A broad-spectrum beta-lactamase TEM-1	
blaSHV-187	100.00	100.00	class A beta-lactamase SHV-187	
mph(E)	100.00	100.00	Mph(E) family macrolide 2'-phosphotransferase	

msr(E)	100.00	100.00	ABC-F type ribosomal protection protein Msr(E)	
armA	100.00	100.00	ArmA family 16S rRNA (guanine(1405)-N(7))-methyltransferase	
arr-2	100.00	100.00	NAD(+)--rifampin ADP-ribosyltransferase Arr-2	
cmlA5	100.00	100.00	chloramphenicol efflux MFS transporter CmlA5	
blaOXA-10	100.00	100.00	oxacillin-hydrolyzing class D beta-lactamase OXA-10	
aadA1	100.00	99.87	ANT(3")-Ia family aminoglycoside nucleotidyltransferase AadA1	
blaCTX-M-2	100.00	100.00	class A extended-spectrum beta-lactamase CTX-M-2	
aadA2	100.00	100.00	ANT(3")-Ia family aminoglycoside nucleotidyltransferase AadA2	
dfrA12	100.00	100.00	trimethoprim-resistant dihydrofolate reductase DfrA12	
fosA6	100.00	99.29	fosfomycin resistance glutathione transferase FosA6	

Table S2. Kleborate-predicted porin alterations in WGS isolates.

Strain	OmpK35 alteration	OmpK36 alteration
K2	OmpK35-7%	OmpK36GD
K5	OmpK35-40%	OmpK36GD
K6	OmpK35-40%	OmpK36GD
K10	OmpK35-0%	OmpK36GD
K16	OmpK35-40%	OmpK36GD
K24	OmpK35-40%	OmpK36GD
K26	-	-
K28	-	-
K29	OmpK35-7%	OmpK36GD
K39	OmpK35-40%	-
K43.1	OmpK35-17%	OmpK36-75%
K43.3	OmpK35-40%	OmpK36GD

Porin alterations in **OmpK35** and **OmpK36** were extracted from Kleborate output for the 12 WGS draft assemblies. Kleborate reports putative truncation/partial hits as a percentage of the expected amino-acid length recovered from the start codon (e.g., **OmpK35–40%** indicates a partial/disrupted locus of ~40% of the expected length). Values close to 0% are consistent with gene loss or severe disruption; however, partial calls can also reflect contig fragmentation in draft assemblies. **OmpK36GD** denotes the Gly-Asp (GD) insertion in the OmpK36 loop 3 (L3) constriction region, which narrows the pore and can reduce β-lactam (including carbapenem) influx. A dash (–) indicates that no porin alteration was flagged by Kleborate for that isolate.

Table S3. K-locus and O-locus assignment of sequenced isolates. Compiled from the TSV file generated by the Kleborate platform.

Code	K_locus	K_type	O_locus	O_type
K10	KL2	K2	O1/O2v2	O1
K16	KL19	K19	O1/O2v2	O2afg
K2	KL2	K2	O1/O2V2	O4
K24	KL19	K19	O1/O2v2	O2a
K26	KL102	KL102	O1/O2v2	O4
K28	KL102	KL102	O1/O2v2	O4
K29	KL2	K2	O1/O2v2	O4
K39	KL19	K19	O1/O2v2	O1
K43.1	KL62	K62	O1/O2v1	O4
K43.3	KL19	K19	O1/O2v2	O2afg
K5	KL19	K19	O1/O2v2	O2a
K6	KL19	K19	O1/O2v2	O4

Table S4. Clinical and epidemiological characterization of 26 patients included in this study.

**Age (years)**		**n (%)**
	Mean ± SD (years)	59 ± 16
	< 45 years	7 (27)
	45-75 years	13 (50)
	> 75 years	6 (23)
**Sex**		**n (%)**
	Male	14 (54)
	Female	12 (46)
**Prior hospitalization**		**n (%)**
	Same facility	3 (50)
	Different facility	3 (50)
**Length of stay (days)**		
	Mean ± SD	85 ± 60
**Originating unit**		**n (%)**
	ICU-2C	31
	ICU-3A	3
	IMCU	3
	ISCU	5
	Acute Stroke Unit	1
	Internal Medicine Unit	1
	Emergency Room	1
**Specimen Source**		**n (%)**
	Endotracheal Aspirate	5 (11)
	Rectal Swab	27 (60)
	Blood Culture	2 (4)
	Urine Culture	4 (9)
	Oropharyngeal Aspirate	4 (9)
	IAC	1 (2)
	Fomite	1 (2)
	Ascitic fluid	1 (2)
**Comorbidities**		**n (%)**
	Arterial hypertension	12 (46)
	Type 2 diabetes mellitus	5 (19)
	Chronic kidney disease	5 (19)
	Dyslipidemia	1 (4)
	Obesity	1 (4)
	Hypothyroidism	1 (4)
	Stroke	3 (12)
	Coronary artery disease	1 (4)
	Chronic liver disease	4 (15)

Chronic GI conditions	2 (8)
COPD	3 (12)
Asthma	1 (4)
Immunodeficiencies	2 (8)
Cancer	3 (12)
Substance abuse	2 (8)
**Invasive Procedures**	**n (%)**
Surgery	13 (50)
Enteroscopy	5 (19)
Bronchoalveolar lavage	2 (8)
**Invasive Medical Devices**	**n (%)**
Endotracheal/tracheostomy tube	23 (88)
CVC/PICCline	25 (96)
Drains / Ostomies	7 (27)
Indwelling urinary catheter	25 (96)
**Antibiotic Treatment**	**n (%)**
Yes	26 (100)
No	0 (0)
**Outcome**	**n (%)**
Survived	18 (69)
Died	8 (31)

Abbreviations: SD, Standard deviation; ICU, Intensive care unit; IMCU, Intermediate medical care unit; ISCU, Intermediate surgical care unit; IAC, Intra-abdominal collection; GI, Gastrointestinal; COPD, Chronic obstructive pulmonary disease; CVC, Central venous catheter.

Table S5. Extract of Fisher's exact test results. Statistical significance level: *p*-value < 0.05. Compiled from the CSV file generated in RStudio, version 2024.09.0+375.

Factor	*blaTEM (-)*	*blaTEM (+)*	*p-value*
Surgery	0 (0%)	20 (51%)	0.053
Bronchoalveolar lavage	1 (20%)	4 (10%)	0.47
Enteroscopy	3 (60%)	6 (15%)	0.050

Table S6. Antibiotics used for susceptibility testing.

Antibiotic family	Antibiotic name	Abbreviations
Cephalosporins	Cefazolin	KZ
	Ceftazidime	CAZ
	Cefotaxime	CTX
	Ceftazidime/Clavulanic acid (for ESBL test)	CAL
	Cefotaxime /Clavulanic acid (for ESBL test)	CLT
Fluoroquinolones	Ciprofloxacin	CIP
	Levofloxacin	LEV
Sulfonamides	Trimethoprim/Sulfamethoxazole	SXT
Aminoglycosides	Amikacin	AK
	Gentamicin	CN
Penicillins	Ampicillin/Sulbactam	SAM
	Piperacillin/Tazobactam	TZP
Carbapenems	Imipenem	IMI
	Ertapenem	ETP
	Meropenem	MEM
Tetracyclines	Tetracycline	TE

Table S7. Primers for carbapenemase gene detection (adapted from Candan & Aksöz, 2015; Poirel *et al*., 2011).

Primers	Sequence (5' a 3')	Amplicon size
*blaKPC*_F	CGTCTAGTTCTGCTGTCTTG	798 pb
*blaKPC*_R	CTTGTCATCCTTGTTAGGCG	
*blaNDM*_F	GGTTTGGCGATCTGGTTTTC	621 pb
*blaNDM*_R	CGGAATGGCTCATCACGATC	
*blaOXA48*_F	GCGTGGTTAAGGATGAACAC	438 pb
*blaOXA48*_R	CATCAAGTTCAACCCAACCG	
*blaIMP*_F	GGAATAGAGTGGCTTAAYTCTC	232 pb
*blaIMP*_R	GGTTTAAYAAAACAACCACC	
*blaVIM*_F	GATGGTGTTTGGTCGCATA	390 pb
*blaVIM*_R	CGAATGCGCAGCACCAG	

Table S8. Primers for ESBL gene detection (adapted from Trung *et al*., 2015).

**Primers**	**Sequences (5' a 3')**	**Amplicon size**
*blaCTX-M*_F	ATGTGCAGYACCAGTAARGTKATGGC	590 pb
*blaCTX-M*_R	GGTRAARTARGTSACCAGAAYCAGCGG	
*blaTEM*_F	TCGCCGCATACACTATTCTCAGAATGAC	422 pb
*blaTEM*_R	CAGCAATAAACCAGCCAGCCGGAAG	
*blaSHV*_F	TGTATTATCTC(C/T)CTGTTAGCC(A/G)CCCTG	739 pb
*blaSHV*_R	GCTCTGCTTTGTTATTCGGGCCAAGC	

Table S9. Primers used in this study for the detection frequency of genes identified as virulence factors in *K. pneumoniae* (adapted from Compain *et al*., 2014).

Primer	Sequence (5' to 3') |	Gene Product/Function	Amplicon Size
*ybtS*_F	GACGGAAACAGCACGGTAAA	Siderophore (yersiniabactin)	242 pb
*ybtS*_R	GAGCATAATAAGGCGAAAGA		
*entB*_F	GTCAACTGGGCCTTTGAGCCGTC	Siderophore (enterobactin)	400 pb
*entB*_R	TATGGGCGTAAACGCCGGTGAT		
*iutA*_F	GGGAAAGGCTTCTCTGCCAT	Siderophore (aerobactin)	920 pb
*iutA*_R	TTATTCGCCACCACGCTCTT		
*kfu*_F	GGCCTTTGTCCAGAGCTACG	Iron transport and phosphotransferase function	638 pb
*kfu*_R	GGGTCTGGCGCAGAGTATGC		
*mrkD*_F	AAGCTATCGCTGTACTTCCGGCA	Type 3 fimbrial adhesin	340 pb
*mrkD*_R	GGCGTTGGCGCTCAGATAGG		
*allS*_F	CATTACGCACCTTTGTCAGC	Allantoin metabolism	764 pb
*allS*_R	GAATGTGTCGGCGATCAGCTT		
*rmpA*_F	CATAAGAGTATTGGTTGACAG	Regulator of mucoid phenotype A	461 pb
*rmpA*_R	CTTGCATGAGCCATCTTTCA		
*k2*_F	CAACCATGGTGGTCGATTAG	Capsular serotype K2	531 pb
*k2*_R	TGGTAGCCATATCCCTTTGG		
*magA*_F	GGTGCTCTTTACATCATTGC	Capsular serotype K1	1238 pb
*magA*_R	GCAATGGCCATTTGCGTTAG		

Table S10. RefSeq genomes included in the regional *Klebsiella pneumoniae* phylogenetic analyses. Genomes were retrieved from the NCBI RefSeq database. "ID" corresponds to the file/label used in downstream analyses (e.g., FASTA filename), whereas "Assembly accession" corresponds to the RefSeq assembly accession. cgLIN codes were assigned using the cgMLST-based hierarchical nomenclature framework (Hennart et al., 2022).

ID	Assembly accession	Country	cgLIN code	Species
GCF_000316265.2_MTE1_ImprovedAssembly_genomic	GCF_000316265.2	Argentina	0_0_1_1_0_0_0_0_0_0	*Klebsiella pneumoniae*
GCF_000512165.1_ASM51216v1_genomic	GCF_000512165.1	Brazil	0_0_152_0_0_0_1_0_0_0	*Klebsiella pneumoniae*
GCF_000807515.1_ASM80751v2_genomic	GCF_000807515.1	Colombia	0_0_197_0_25_0_0_0_0_0	*Klebsiella pneumoniae*
GCF_001701895.1_ASM170189v1_genomic	GCF_001701895.1	Brazil	0_0_105_0_0_0_1_1_0_0	*Klebsiella pneumoniae*
GCF_001701915.1_ASM170191v1_genomic	GCF_001701915.1	Brazil	0_0_105_1_1_1_7_0_0_0	*Klebsiella pneumoniae*
GCF_001718115.2_ASM171811v2_genomic	GCF_001718115.2	Brazil	0_0_105_0_11_0_0_0_1_0	*Klebsiella pneumoniae*
GCF_001718175.1_ASM171817v1_genomic	GCF_001718175.1	Brazil	0_0_105_0_11_0_0_8_0_0	*Klebsiella pneumoniae*
GCF_001907955.1_ASM190795v1_genomic	GCF_001907955.1	Brazil	0_0_407_0_0_0_0_1_0_0	*Klebsiella pneumoniae*
GCF_002104175.1_ASM210417v1_genomic	GCF_002104175.1	Brazil	0_0_105_0_11_0_6_0_0_0	*Klebsiella pneumoniae*
GCF_002104185.1_ASM210418v1_genomic	GCF_002104185.1	Brazil	0_0_105_6_0_0_69_0_1_0	*Klebsiella pneumoniae*
GCF_002104215.1_ASM210421v1_genomic	GCF_002104215.1	Brazil	0_0_105_0_0_0_1_2_0_0	*Klebsiella pneumoniae*
GCF_002104255.1_ASM210425v1_genomic	GCF_002104255.1	Brazil	0_0_105_0_11_0_12_0_0_0	*Klebsiella pneumoniae*
GCF_002104265.1_ASM210426v1_genomic	GCF_002104265.1	Brazil	0_0_105_6_0_0_69_0_0_0	*Klebsiella pneumoniae*
GCF_002104295.1_ASM210429v1_genomic	GCF_002104295.1	Brazil	0_0_105_6_0_0_69_0_1_0	*Klebsiella pneumoniae*
GCF_002104315.1_ASM210431v1_genomic	GCF_002104315.1	Brazil	0_0_105_0_0_0_2_0_0_0	*Klebsiella pneumoniae*
GCF_002104355.1_ASM210435v1_genomic	GCF_002104355.1	Brazil	0_0_105_0_0_0_10_0_0_0	*Klebsiella pneumoniae*
GCF_002104365.1_ASM210436v1_genomic	GCF_002104365.1	Brazil	0_0_105_0_11_0_12_0_1_0	*Klebsiella pneumoniae*
GCF_002119885.1_ASM211988v1_genomic	GCF_002119885.1	Colombia	0_0_105_6_0_0_17_58_0_0	*Klebsiella pneumoniae*
GCF_002119895.1_ASM211989v1_genomic	GCF_002119895.1	Colombia	0_0_105_6_0_0_26_0_0_0	*Klebsiella pneumoniae*
GCF_002119955.1_ASM211995v1_genomic	GCF_002119955.1	Colombia	0_0_1_1_0_1_1_0_0_2	*Klebsiella pneumoniae*
GCF_002120065.1_ASM212006v1_genomic	GCF_002120065.1	Colombia	0_0_105_6_0_0_77_0_0_0	*Klebsiella pneumoniae*
GCF_002120105.1_ASM212010v1_genomic	GCF_002120105.1	Colombia	0_0_105_6_0_0_17_57_0_0	*Klebsiella pneumoniae*
GCF_002154825.1_ASM215482v1_genomic	GCF_002154825.1	Brazil	0_0_105_0_11_0_5_0_0_0	*Klebsiella pneumoniae*
GCF_002157345.1_ASM215734v1_genomic	GCF_002157345.1	Brazil	0_0_407_0_0_0_0_0_1_0	*Klebsiella pneumoniae*
GCF_002192975.1_ASM219297v1_genomic	GCF_002192975.1	Brazil	0_0_105_0_11_0_6_0_0_0	*Klebsiella pneumoniae*
GCF_002241035.1_ASM224103v1_genomic	GCF_002241035.1	Brazil	0_0_407_0_0_0_0_0_0_0	*Klebsiella pneumoniae*
GCF_002251715.1_ASM225171v1_genomic	GCF_002251715.1	Brazil	0_0_105_1_1_1_4_1_0_0	*Klebsiella pneumoniae*
GCF_002283495.1_ASM228349v1_genomic	GCF_002283495.1	Colombia	0_0_369_0_0_0_0_0_20_0	*Klebsiella pneumoniae*
GCF_002529065.1_ASM252906v1_genomic	GCF_002529065.1	Brazil	0_0_105_0_0_0_7_0_0_0	*Klebsiella pneumoniae*
GCF_002529075.1_ASM252907v1_genomic	GCF_002529075.1	Brazil	0_0_105_0_0_0_1_0_1_0	*Klebsiella pneumoniae*

GCF_002631045.1_ASM263104v1_genomic	GCF_002631045.1	Brazil	0_0_152_0_0_1_0_0_0_0	*Klebsiella pneumoniae*
GCF_002834285.1_ASM283428v1_genomic	GCF_002834285.1	Peru	0_0_105_0_11_2_0_0_0_0	*Klebsiella pneumoniae*
GCF_002850795.1_ASM285079v1_genomic	GCF_002850795.1	Colombia	0_0_419_0_0_0_0_0_0_0	*Klebsiella pneumoniae*
GCF_002850805.1_ASM285080v1_genomic	GCF_002850805.1	Colombia	0_0_1_1_0_1_1_0_0_0	*Klebsiella pneumoniae*
GCF_002850835.1_ASM285083v1_genomic	GCF_002850835.1	Colombia	0_0_108_3_0_0_0_0_0_0	*Klebsiella pneumoniae*
GCF_002850845.1_ASM285084v1_genomic	GCF_002850845.1	Colombia	0_0_13_11_0_0_0_0_0_0	*Klebsiella pneumoniae*
GCF_002850875.1_ASM285087v1_genomic	GCF_002850875.1	Colombia	0_0_22_30_0_0_0_0_0_0	*Klebsiella pneumoniae*
GCF_002850885.1_ASM285088v1_genomic	GCF_002850885.1	Colombia	0_0_1_1_0_1_1_0_0_3	*Klebsiella pneumoniae*
GCF_002850915.1_ASM285091v1_genomic	GCF_002850915.1	Colombia	0_0_107_0_1_4_0_0_0_0	*Klebsiella pneumoniae*
GCF_002850935.1_ASM285093v1_genomic	GCF_002850935.1	Colombia	0_0_1_1_0_1_1_0_0_1	*Klebsiella pneumoniae*
GCF_002850955.1_ASM285095v1_genomic	GCF_002850955.1	Colombia	0_0_1_1_9_0_0_0_0_0	*Klebsiella pneumoniae*
GCF_002850965.1_ASM285096v1_genomic	GCF_002850965.1	Colombia	0_0_147_1_0_4_0_0_0_0	*Klebsiella pneumoniae*
GCF_002850995.1_ASM285099v1_genomic	GCF_002850995.1	Colombia	0_0_127_1_0_0_0_0_0_0	*Klebsiella pneumoniae*
GCF_002851015.1_ASM285101v1_genomic	GCF_002851015.1	Colombia	0_0_0_0_16_0_0_0_0_0	*Klebsiella pneumoniae*
GCF_002851035.1_ASM285103v1_genomic	GCF_002851035.1	Colombia	0_0_105_6_0_0_17_114_0_0	*Klebsiella pneumoniae*
GCF_002851055.1_ASM285105v1_genomic	GCF_002851055.1	Colombia	0_0_123_1_0_0_0_0_0_0	*Klebsiella pneumoniae*
GCF_002851115.1_ASM285111v1_genomic	GCF_002851115.1	Colombia	0_0_276_0_0_0_0_0_0_0	*Klebsiella pneumoniae*
GCF_002851135.1_ASM285113v1_genomic	GCF_002851135.1	Colombia	0_0_111_0_5_0_0_0_0_0	*Klebsiella pneumoniae*
GCF_002851155.1_ASM285115v1_genomic	GCF_002851155.1	Colombia	0_0_152_0_2_0_0_0_0_0	*Klebsiella pneumoniae*
GCF_002851175.1_ASM285117v1_genomic	GCF_002851175.1	Colombia	0_0_105_10_0_0_0_0_0_0	*Klebsiella pneumoniae*
GCF_002851195.1_ASM285119v1_genomic	GCF_002851195.1	Colombia	0_0_98_0_1_0_0_0_0_0	*Klebsiella pneumoniae*
GCF_002851215.1_ASM285121v1_genomic	GCF_002851215.1	Colombia	0_0_300_1_0_0_0_0_0_0	*Klebsiella pneumoniae*
GCF_002851245.1_ASM285124v1_genomic	GCF_002851245.1	Colombia	0_0_104_0_0_0_0_0_0_0	*Klebsiella pneumoniae*
GCF_002851275.1_ASM285127v1_genomic	GCF_002851275.1	Colombia	0_0_94_2_1_0_0_0_0_0	*Klebsiella pneumoniae*
GCF_002851335.1_ASM285133v1_genomic	GCF_002851335.1	Colombia	0_0_41_0_0_0_0_0_0_0	*Klebsiella pneumoniae*
GCF_002851345.1_ASM285134v1_genomic	GCF_002851345.1	Colombia	0_0_84_0_8_0_0_9_0_0	*Klebsiella pneumoniae*
GCF_002851375.1_ASM285137v1_genomic	GCF_002851375.1	Colombia	0_0_105_0_6_0_0_0_0_0	*Klebsiella pneumoniae*
GCF_002851395.1_ASM285139v1_genomic	GCF_002851395.1	Colombia	0_0_107_2_0_0_0_0_0_0	*Klebsiella pneumoniae*
GCF_002851435.1_ASM285143v1_genomic	GCF_002851435.1	Colombia	0_0_1_1_0_1_1_0_2_0	*Klebsiella pneumoniae*
GCF_002851455.1_ASM285145v1_genomic	GCF_002851455.1	Colombia	0_0_22_24_1_0_0_0_0_0	*Klebsiella pneumoniae*
GCF_002851475.1_ASM285147v1_genomic	GCF_002851475.1	Colombia	0_0_137_0_1_0_0_0_0_0	*Klebsiella pneumoniae*
GCF_002851495.1_ASM285149v1_genomic	GCF_002851495.1	Colombia	0_0_46_8_0_0_0_0_0_0	*Klebsiella pneumoniae*
GCF_002851515.1_ASM285151v1_genomic	GCF_002851515.1	Colombia	0_0_250_0_4_0_0_0_0_0	*Klebsiella pneumoniae*
GCF_002851525.1_ASM285152v1_genomic	GCF_002851525.1	Colombia	0_0_361_0_0_0_0_0_0_0	*Klebsiella pneumoniae*

GCF_002851555.1_ASM285155v1_genomic	GCF_002851555.1	Colombia	0_0_1_1_0_1_1_0_2_0	*Klebsiella pneumoniae*
GCF_002851575.1_ASM285157v1_genomic	GCF_002851575.1	Colombia	0_0_260_0_0_0_0_0_0_0	*Klebsiella pneumoniae*
GCF_002851615.1_ASM285161v1_genomic	GCF_002851615.1	Colombia	0_0_240_0_0_0_0_0_0_0	*Klebsiella pneumoniae*
GCF_002853565.1_ASM285356v1_genomic	GCF_002853565.1	Colombia	0_0_67_0_0_0_0_0_0_0	*Klebsiella pneumoniae*
GCF_002853595.1_ASM285359v1_genomic	GCF_002853595.1	Colombia	0_0_220_7_4_0_0_0_0_0	*Klebsiella pneumoniae*
GCF_002853725.1_ASM285372v1_genomic	GCF_002853725.1	Colombia	0_0_47_0_2_0_0_0_0_0	*Klebsiella pneumoniae*
GCF_002853735.1_ASM285373v1_genomic	GCF_002853735.1	Colombia	0_0_131_1_23_0_0_0_0_0	*Klebsiella pneumoniae*
GCF_002853795.1_ASM285379v1_genomic	GCF_002853795.1	Colombia	0_0_237_1_0_1_0_0_0_0	*Klebsiella pneumoniae*
GCF_002853815.1_ASM285381v1_genomic	GCF_002853815.1	Colombia	0_0_22_2_16_0_0_0_0_0	*Klebsiella pneumoniae*
GCF_002853825.1_ASM285382v1_genomic	GCF_002853825.1	Colombia	0_0_22_24_10_0_0_0_0_0	*Klebsiella pneumoniae*
GCF_002853895.1_ASM285389v1_genomic	GCF_002853895.1	Colombia	0_0_29_1_0_0_0_0_0_0	*Klebsiella pneumoniae*
GCF_002853915.1_ASM285391v1_genomic	GCF_002853915.1	Colombia	0_0_334_0_1_0_0_0_0_0	*Klebsiella pneumoniae*
GCF_002853935.1_ASM285393v1_genomic	GCF_002853935.1	Colombia	0_0_194_0_0_0_0_0_0_0	*Klebsiella pneumoniae*
GCF_002853955.1_ASM285395v1_genomic	GCF_002853955.1	Colombia	0_0_23_0_0_0_0_0_0_0	*Klebsiella pneumoniae*
GCF_002853975.1_ASM285397v1_genomic	GCF_002853975.1	Colombia	0_0_1_1_14_0_0_0_0_0	*Klebsiella pneumoniae*
GCF_002854015.1_ASM285401v1_genomic	GCF_002854015.1	Colombia	0_0_1_1_0_1_1_0_1_0	*Klebsiella pneumoniae*
GCF_002854035.1_ASM285403v1_genomic	GCF_002854035.1	Colombia	0_0_116_0_0_0_0_0_0_0	*Klebsiella pneumoniae*
GCF_002854055.1_ASM285405v1_genomic	GCF_002854055.1	Colombia	0_0_108_4_0_0_0_0_0_0	*Klebsiella pneumoniae*
GCF_002854085.1_ASM285408v1_genomic	GCF_002854085.1	Colombia	0_0_98_0_5_0_0_0_0_0	*Klebsiella pneumoniae*
GCF_002854115.1_ASM285411v1_genomic	GCF_002854115.1	Colombia	0_0_253_0_0_0_0_0_0_0	*Klebsiella pneumoniae*
GCF_002854135.1_ASM285413v1_genomic	GCF_002854135.1	Colombia	0_0_28_0_0_0_0_0_0_0	*Klebsiella pneumoniae*
GCF_002854145.1_ASM285414v1_genomic	GCF_002854145.1	Colombia	0_0_253_0_0_0_0_0_0_0	*Klebsiella pneumoniae*
GCF_002854175.1_ASM285417v1_genomic	GCF_002854175.1	Colombia	0_0_238_0_0_0_0_0_0_0	*Klebsiella pneumoniae*
GCF_002854195.1_ASM285419v1_genomic	GCF_002854195.1	Colombia	0_0_197_0_4_0_0_0_1_0	*Klebsiella pneumoniae*
GCF_002854215.1_ASM285421v1_genomic	GCF_002854215.1	Colombia	0_0_0_0_2_1_0_0_0_0	*Klebsiella pneumoniae*
GCF_002854235.1_ASM285423v1_genomic	GCF_002854235.1	Colombia	0_0_105_6_0_0_17_42_0_0	*Klebsiella pneumoniae*
GCF_002854255.1_ASM285425v1_genomic	GCF_002854255.1	Colombia	0_0_28_0_1_0_0_0_0_0	*Klebsiella pneumoniae*
GCF_002854275.1_ASM285427v1_genomic	GCF_002854275.1	Colombia	0_0_105_6_0_0_17_16_8_0	*Klebsiella pneumoniae*
GCF_002854285.1_ASM285428v1_genomic	GCF_002854285.1	Colombia	0_0_22_30_1_0_0_0_0_0	*Klebsiella pneumoniae*
GCF_002854295.1_ASM285429v1_genomic	GCF_002854295.1	Colombia	0_0_105_6_0_0_17_16_0_0	*Klebsiella pneumoniae*
GCF_002854335.1_ASM285433v1_genomic	GCF_002854335.1	Colombia	0_0_105_6_0_0_17_16_9_0	*Klebsiella pneumoniae*
GCF_002854355.1_ASM285435v1_genomic	GCF_002854355.1	Colombia	0_0_84_0_8_0_3_0_0_0	*Klebsiella pneumoniae*
GCF_002854375.1_ASM285437v1_genomic	GCF_002854375.1	Colombia	0_0_105_6_0_0_17_16_7_0	*Klebsiella pneumoniae*
GCF_002854385.1_ASM285438v1_genomic	GCF_002854385.1	Colombia	0_0_105_6_0_0_17_16_0_0	*Klebsiella pneumoniae*

GCF_002854415.1_ASM285441v1_genomic	GCF_002854415.1	Colombia	0_0_105_6_0_0_17_16_2_0	*Klebsiella pneumoniae*
GCF_002854435.1_ASM285443v1_genomic	GCF_002854435.1	Colombia	0_0_105_6_0_0_17_41_0_0	*Klebsiella pneumoniae*
GCF_002854455.1_ASM285445v1_genomic	GCF_002854455.1	Colombia	0_0_105_6_0_0_17_53_1_0	*Klebsiella pneumoniae*
GCF_002854465.1_ASM285446v1_genomic	GCF_002854465.1	Colombia	0_0_105_6_0_0_17_16_0_0	*Klebsiella pneumoniae*
GCF_002854495.1_ASM285449v1_genomic	GCF_002854495.1	Colombia	0_0_105_6_0_0_17_16_0_0	*Klebsiella pneumoniae*
GCF_002854515.1_ASM285451v1_genomic	GCF_002854515.1	Colombia	0_0_197_0_4_0_0_0_0_0	*Klebsiella pneumoniae*
GCF_002854525.1_ASM285452v1_genomic	GCF_002854525.1	Colombia	0_0_105_6_0_0_17_53_1_0	*Klebsiella pneumoniae*
GCF_002854575.1_ASM285457v1_genomic	GCF_002854575.1	Colombia	0_0_1_1_0_1_1_0_0_0	*Klebsiella pneumoniae*
GCF_002854585.1_ASM285458v1_genomic	GCF_002854585.1	Colombia	0_0_158_8_0_0_7_0_0_0	*Klebsiella pneumoniae*
GCF_002854635.1_ASM285463v1_genomic	GCF_002854635.1	Colombia	0_0_158_8_0_0_5_0_0_0	*Klebsiella pneumoniae*
GCF_002854655.1_ASM285465v1_genomic	GCF_002854655.1	Colombia	0_0_197_0_0_0_0_2_0_0	*Klebsiella pneumoniae*
GCF_002854675.1_ASM285467v1_genomic	GCF_002854675.1	Colombia	0_0_22_24_7_0_0_0_0_0	*Klebsiella pneumoniae*
GCF_002854695.1_ASM285469v1_genomic	GCF_002854695.1	Colombia	0_0_84_0_8_0_0_9_0_0	*Klebsiella pneumoniae*
GCF_002854715.1_ASM285471v1_genomic	GCF_002854715.1	Colombia	0_0_84_0_8_0_0_9_0_0	*Klebsiella pneumoniae*
GCF_002854735.1_ASM285473v1_genomic	GCF_002854735.1	Colombia	0_0_105_6_0_0_17_16_1_0	*Klebsiella pneumoniae*
GCF_002854745.1_ASM285474v1_genomic	GCF_002854745.1	Colombia	0_0_94_1_2_0_0_0_0_0	*Klebsiella pneumoniae*
GCF_002854815.1_ASM285481v1_genomic	GCF_002854815.1	Colombia	0_0_197_0_0_0_0_1_0_0	*Klebsiella pneumoniae*
GCF_002854835.1_ASM285483v1_genomic	GCF_002854835.1	Colombia	0_0_23_1_4_0_0_0_0_0	*Klebsiella pneumoniae*
GCF_002854855.1_ASM285485v1_genomic	GCF_002854855.1	Colombia	0_0_52_4_1_0_0_0_0_0	*Klebsiella pneumoniae*
GCF_002854865.1_ASM285486v1_genomic	GCF_002854865.1	Colombia	0_0_105_6_0_0_17_16_6_0	*Klebsiella pneumoniae*
GCF_002854895.1_ASM285489v1_genomic	GCF_002854895.1	Colombia	0_0_105_6_0_0_17_16_5_0	*Klebsiella pneumoniae*
GCF_002854915.1_ASM285491v1_genomic	GCF_002854915.1	Colombia	0_0_98_0_5_0_0_0_0_0	*Klebsiella pneumoniae*
GCF_002854935.1_ASM285493v1_genomic	GCF_002854935.1	Colombia	0_0_23_0_0_0_0_0_0_0	*Klebsiella pneumoniae*
GCF_002854955.1_ASM285495v1_genomic	GCF_002854955.1	Colombia	0_0_84_0_8_0_3_0_0_0	*Klebsiella pneumoniae*
GCF_002854975.1_ASM285497v1_genomic	GCF_002854975.1	Colombia	0_0_105_6_0_0_17_16_0_0	*Klebsiella pneumoniae*
GCF_002854995.1_ASM285499v1_genomic	GCF_002854995.1	Colombia	0_0_105_6_0_0_17_16_4_0	*Klebsiella pneumoniae*
GCF_002855015.1_ASM285501v1_genomic	GCF_002855015.1	Colombia	0_0_410_0_3_0_0_0_0_0	*Klebsiella pneumoniae*
GCF_002855035.1_ASM285503v1_genomic	GCF_002855035.1	Colombia	0_0_105_6_0_0_17_16_0_0	*Klebsiella pneumoniae*
GCF_002855045.1_ASM285504v1_genomic	GCF_002855045.1	Colombia	0_0_105_6_0_0_17_16_0_0	*Klebsiella pneumoniae*
GCF_002855075.1_ASM285507v1_genomic	GCF_002855075.1	Colombia	0_0_105_6_0_0_17_16_3_0	*Klebsiella pneumoniae*
GCF_002855115.1_ASM285511v1_genomic	GCF_002855115.1	Colombia	0_0_105_6_0_0_17_16_0_0	*Klebsiella pneumoniae*
GCF_002855125.1_ASM285512v1_genomic	GCF_002855125.1	Colombia	0_0_105_6_0_0_17_16_0_0	*Klebsiella pneumoniae*
GCF_002855155.1_ASM285515v1_genomic	GCF_002855155.1	Colombia	0_0_108_3_0_0_0_0_0_0	*Klebsiella pneumoniae*
GCF_002855175.1_ASM285517v1_genomic	GCF_002855175.1	Colombia	0_0_22_2_19_0_0_0_0_0	*Klebsiella pneumoniae*

GCF_002855195.1_ASM285519v1_genomic	GCF_002855195.1	Colombia	0_0_105_6_0_0_17_16_0_0	*Klebsiella pneumoniae*
GCF_002855215.1_ASM285521v1_genomic	GCF_002855215.1	Colombia	0_0_105_6_0_0_17_56_0_0	*Klebsiella pneumoniae*
GCF_002855255.1_ASM285525v1_genomic	GCF_002855255.1	Colombia	0_0_1_1_0_1_1_0_0_0	*Klebsiella pneumoniae*
GCF_002855275.1_ASM285527v1_genomic	GCF_002855275.1	Colombia	0_0_105_6_0_0_87_0_0_0	*Klebsiella pneumoniae*
GCF_002855295.1_ASM285529v1_genomic	GCF_002855295.1	Colombia	0_0_1_1_0_1_1_0_0_0	*Klebsiella pneumoniae*
GCF_002855315.1_ASM285531v1_genomic	GCF_002855315.1	Colombia	0_0_84_0_8_0_0_9_1_0	*Klebsiella pneumoniae*
GCF_002855375.1_ASM285537v1_genomic	GCF_002855375.1	Colombia	0_0_48_0_0_0_0_0_0_0	*Klebsiella pneumoniae*
GCF_002855395.1_ASM285539v1_genomic	GCF_002855395.1	Colombia	0_0_22_24_7_0_0_0_0_0	*Klebsiella pneumoniae*
GCF_002887435.2_ASM288743v2_genomic	GCF_002887435.2	Brazil	0_0_105_0_11_0_0_13_0_0	*Klebsiella pneumoniae*
GCF_002929175.1_ASM292917v1_genomic	GCF_002929175.1	Brazil	0_0_6_0_1_0_0_0_0_0	*Klebsiella pneumoniae*
GCF_002951555.1_ASM295155v1_genomic	GCF_002951555.1	Brazil	0_0_105_1_1_1_0_15_0_0	*Klebsiella pneumoniae*
GCF_002951595.1_ASM295159v1_genomic	GCF_002951595.1	Brazil	0_0_105_1_1_1_0_1_0_0	*Klebsiella pneumoniae*
GCF_003095455.1_ASM309545v1_genomic	GCF_003095455.1	Brazil	0_0_105_6_0_90_0_0_0_0	*Klebsiella pneumoniae*
GCF_003095475.1_ASM309547v1_genomic	GCF_003095475.1	Brazil	0_0_388_1_32_0_0_0_0_0	*Klebsiella pneumoniae*
GCF_003095495.1_ASM309549v1_genomic	GCF_003095495.1	Brazil	0_0_105_6_0_0_373_0_0_0	*Klebsiella pneumoniae*
GCF_003095515.1_ASM309551v1_genomic	GCF_003095515.1	Brazil	0_0_105_6_0_0_374_0_0_0	*Klebsiella pneumoniae*
GCF_003095615.1_ASM309561v1_genomic	GCF_003095615.1	Brazil	0_0_152_0_14_0_0_0_0_0	*Klebsiella pneumoniae*
GCF_003097475.1_ASM309747v1_genomic	GCF_003097475.1	Brazil	0_0_366_0_0_0_0_0_0_0	*Klebsiella pneumoniae*
GCF_003194695.1_ASM319469v1_genomic	GCF_003194695.1	Brazil	0_0_369_0_0_0_0_20_0_0	*Klebsiella pneumoniae*
GCF_003194705.1_ASM319470v1_genomic	GCF_003194705.1	Brazil	0_0_369_0_0_0_0_6_0_0	*Klebsiella pneumoniae*
GCF_003284835.1_ASM328483v1_genomic	GCF_003284835.1	Brazil	0_0_105_0_0_0_0_0_0_0	*Klebsiella pneumoniae*
GCF_003318315.1_ASM331831v1_genomic	GCF_003318315.1	Brazil	0_0_109_1_19_0_0_0_0_0	*Klebsiella pneumoniae*
GCF_003321095.1_ASM332109v1_genomic	GCF_003321095.1	Brazil	0_0_22_27_5_0_0_0_0_0	*Klebsiella pneumoniae*
GCF_003321135.1_ASM332113v1_genomic	GCF_003321135.1	Brazil	0_0_105_6_0_0_69_0_2_0	*Klebsiella pneumoniae*
GCF_003321155.1_ASM332115v1_genomic	GCF_003321155.1	Brazil	0_0_105_0_0_0_0_1_0_0	*Klebsiella pneumoniae*
GCF_003321195.1_ASM332119v1_genomic	GCF_003321195.1	Brazil	0_0_105_0_25_0_0_0_0_0	*Klebsiella pneumoniae*
GCF_003321215.1_ASM332121v1_genomic	GCF_003321215.1	Brazil	0_0_105_0_0_0_0_2_0_0	*Klebsiella pneumoniae*
GCF_003321225.1_ASM332122v1_genomic	GCF_003321225.1	Brazil	0_0_105_0_24_0_0_0_0_0	*Klebsiella pneumoniae*
GCF_003321235.1_ASM332123v1_genomic	GCF_003321235.1	Brazil	0_0_105_0_24_0_3_0_0_0	*Klebsiella pneumoniae*
GCF_003321255.1_ASM332125v1_genomic	GCF_003321255.1	Brazil	0_0_105_0_11_0_12_0_2_0	*Klebsiella pneumoniae*
GCF_003321295.1_ASM332129v1_genomic	GCF_003321295.1	Brazil	0_0_105_0_0_0_8_0_0_0	*Klebsiella pneumoniae*
GCF_003321305.1_ASM332130v1_genomic	GCF_003321305.1	Brazil	0_0_105_0_11_0_6_0_1_0	*Klebsiella pneumoniae*
GCF_003321315.1_ASM332131v1_genomic	GCF_003321315.1	Brazil	0_0_105_0_0_0_9_0_0_0	*Klebsiella pneumoniae*
GCF_003321335.1_ASM332133v1_genomic	GCF_003321335.1	Brazil	0_0_105_0_11_0_6_0_0_0	*Klebsiella pneumoniae*

GCF_003321375.1_ASM332137v1_genomic	GCF_003321375.1	Brazil	0_0_105_0_11_0_6_0_0_0	*Klebsiella pneumoniae*
GCF_003321385.1_ASM332138v1_genomic	GCF_003321385.1	Brazil	0_0_105_0_11_0_0_12_0_0	*Klebsiella pneumoniae*
GCF_003325525.1_ASM332552v1_genomic	GCF_003325525.1	Brazil	0_0_152_0_1_0_0_0_0_0	*Klebsiella pneumoniae*
GCF_003326285.1_ASM332628v1_genomic	GCF_003326285.1	Brazil	0_0_105_6_7_0_0_0_0_0	*Klebsiella pneumoniae*
GCF_003326295.1_ASM332629v1_genomic	GCF_003326295.1	Brazil	0_0_105_0_0_3_0_0_0_0	*Klebsiella pneumoniae*
GCF_003326305.1_ASM332630v1_genomic	GCF_003326305.1	Brazil	0_0_105_6_0_0_70_0_0_0	*Klebsiella pneumoniae*
GCF_003326315.1_ASM332631v1_genomic	GCF_003326315.1	Brazil	0_0_105_6_0_0_69_1_0_0	*Klebsiella pneumoniae*
GCF_003326365.1_ASM332636v1_genomic	GCF_003326365.1	Brazil	0_0_105_6_0_15_0_0_0_0	*Klebsiella pneumoniae*
GCF_003326375.1_ASM332637v1_genomic	GCF_003326375.1	Brazil	0_0_105_1_2_0_0_0_0_0	*Klebsiella pneumoniae*
GCF_003326385.1_ASM332638v1_genomic	GCF_003326385.1	Brazil	0_0_105_0_0_10_0_0_0_0	*Klebsiella pneumoniae*
GCF_003326395.1_ASM332639v1_genomic	GCF_003326395.1	Brazil	0_0_22_27_3_0_0_0_0_0	*Klebsiella pneumoniae*
GCF_003327585.1_ASM332758v1_genomic	GCF_003327585.1	Brazil	0_0_105_0_11_0_0_4_0_0	*Klebsiella pneumoniae*
GCF_003574335.1_ASM357433v1_genomic	GCF_003574335.1	Uruguay	0_0_105_6_0_0_0_93_0_0	*Klebsiella pneumoniae*
GCF_003576025.1_ASM357602v1_genomic	GCF_003576025.1	Brazil	0_0_105_0_11_0_10_0_0_0	*Klebsiella pneumoniae*
GCF_004284565.1_ASM428456v1_genomic	GCF_004284565.1	Brazil	0_0_105_0_11_3_0_0_0_0	*Klebsiella pneumoniae*
GCF_005222595.1_ASM522259v1_genomic	GCF_005222595.1	Brazil	0_0_0_0_2_0_163_0_0_0	*Klebsiella pneumoniae*
GCF_006152045.1_ASM615204v1_genomic	GCF_006152045.1	Brazil	0_0_137_6_0_0_0_0_3_0	*Klebsiella pneumoniae*
GCF_006152055.2_ASM615205v2_genomic	GCF_006152055.2	Brazil	0_0_751_0_0_0_0_0_0_0	*Klebsiella pneumoniae*
GCF_006152075.1_ASM615207v1_genomic	GCF_006152075.1	Brazil	0_0_137_6_0_0_0_0_3_1	*Klebsiella pneumoniae*
GCF_006335135.1_ASM633513v1_genomic	GCF_006335135.1	Chile	0_0_105_6_0_0_375_0_0_0	*Klebsiella pneumoniae*
GCF_006517515.1_ASM651751v1_genomic	GCF_006517515.1	Brazil	0_0_105_0_9_0_0_28_1_0	*Klebsiella pneumoniae*
GCF_008123385.1_ASM812338v1_genomic	GCF_008123385.1	Chile	0_0_137_8_0_0_1_0_0_0	*Klebsiella pneumoniae*
GCF_009184435.1_ASM918443v1_genomic	GCF_009184435.1	Brazil	0_0_944_0_0_0_0_0_0_0	*Klebsiella pneumoniae*
GCF_009184445.1_ASM918444v1_genomic	GCF_009184445.1	Brazil	0_0_122_28_0_0_0_0_0_0	*Klebsiella pneumoniae*
GCF_009184455.1_ASM918445v1_genomic	GCF_009184455.1	Brazil	0_0_520_0_2_0_0_0_0_0	*Klebsiella pneumoniae*
GCF_009758275.2_ASM975827v2_genomic	GCF_009758275.2	Brazil	0_0_197_0_74_0_3_0_0_0	*Klebsiella pneumoniae*
GCF_009898155.1_ASM989815v1_genomic	GCF_009898155.1	Brazil	0_0_197_0_25_0_0_0_15_0	*Klebsiella pneumoniae*
GCF_009928565.1_ASM992856v1_genomic	GCF_009928565.1	Argentina	0_0_105_6_0_0_378_0_0_0	*Klebsiella pneumoniae*
GCF_009928615.1_ASM992861v1_genomic	GCF_009928615.1	Argentina	0_0_0_0_7_3_0_0_0_0	*Klebsiella pneumoniae*
GCF_010671585.1_ASM1067158v1_genomic	GCF_010671585.1	Chile	0_0_137_8_0_0_0_1_0_0	*Klebsiella pneumoniae*
GCF_011008735.1_ASM1100873v1_genomic	GCF_011008735.1	Chile	0_0_388_0_1_0_14_0_0_0	*Klebsiella pneumoniae*
GCF_011008795.1_ASM1100879v1_genomic	GCF_011008795.1	Chile	0_0_105_0_20_1_0_0_0_0	*Klebsiella pneumoniae*
GCF_011008835.1_ASM1100883v1_genomic	GCF_011008835.1	Chile	0_0_388_0_1_0_9_0_9_0	*Klebsiella pneumoniae*
GCF_011008895.1_ASM1100889v1_genomic	GCF_011008895.1	Chile	0_0_388_0_1_0_9_12_0_0	*Klebsiella pneumoniae*

GCF_011037215.1_ASM1103721v1_genomic	GCF_011037215.1	Brazil	0_0_105_0_75_0_0_0_0_0	*Klebsiella pneumoniae*
GCF_011037225.1_ASM1103722v1_genomic	GCF_011037225.1	Brazil	0_0_105_1_1_1_19_0_0_0	*Klebsiella pneumoniae*
GCF_011037245.1_ASM1103724v1_genomic	GCF_011037245.1	Brazil	0_0_105_1_1_1_19_0_1_0	*Klebsiella pneumoniae*
GCF_011037275.1_ASM1103727v1_genomic	GCF_011037275.1	Brazil	0_0_105_1_1_1_19_0_0_0	*Klebsiella pneumoniae*
GCF_011037295.1_ASM1103729v1_genomic	GCF_011037295.1	Brazil	0_0_105_1_1_1_19_0_0_0	*Klebsiella pneumoniae*
GCF_011037315.1_ASM1103731v1_genomic	GCF_011037315.1	Brazil	0_0_105_1_1_1_19_0_0_0	*Klebsiella pneumoniae*
GCF_011037335.1_ASM1103733v1_genomic	GCF_011037335.1	Brazil	0_0_105_1_1_7_0_0_0_0	*Klebsiella pneumoniae*
GCF_011037345.1_ASM1103734v1_genomic	GCF_011037345.1	Brazil	0_0_105_1_1_1_0_0_3_0	*Klebsiella pneumoniae*
GCF_011037375.1_ASM1103737v1_genomic	GCF_011037375.1	Brazil	0_0_105_1_1_1_19_0_0_0	*Klebsiella pneumoniae*
GCF_011037395.1_ASM1103739v1_genomic	GCF_011037395.1	Brazil	0_0_105_1_1_7_0_0_1_0	*Klebsiella pneumoniae*
GCF_011037415.1_ASM1103741v1_genomic	GCF_011037415.1	Brazil	0_0_105_1_1_1_19_0_0_0	*Klebsiella pneumoniae*
GCF_011037435.1_ASM1103743v1_genomic	GCF_011037435.1	Brazil	0_0_105_1_1_1_19_0_0_0	*Klebsiella pneumoniae*
GCF_011037455.1_ASM1103745v1_genomic	GCF_011037455.1	Brazil	0_0_105_0_0_0_1_8_0_0	*Klebsiella pneumoniae*
GCF_011037475.1_ASM1103747v1_genomic	GCF_011037475.1	Brazil	0_0_105_0_9_0_0_28_1_0	*Klebsiella pneumoniae*
GCF_011037495.1_ASM1103749v1_genomic	GCF_011037495.1	Brazil	0_0_105_0_0_0_1_9_0_0	*Klebsiella pneumoniae*
GCF_011037515.1_ASM1103751v1_genomic	GCF_011037515.1	Brazil	0_0_105_0_0_0_1_10_0_0	*Klebsiella pneumoniae*
GCF_011037535.1_ASM1103753v1_genomic	GCF_011037535.1	Brazil	0_0_105_0_0_0_1_1_2_0	*Klebsiella pneumoniae*
GCF_011037555.1_ASM1103755v1_genomic	GCF_011037555.1	Brazil	0_0_105_0_9_0_0_28_1_0	*Klebsiella pneumoniae*
GCF_011037575.1_ASM1103757v1_genomic	GCF_011037575.1	Brazil	0_0_105_0_9_0_0_28_1_0	*Klebsiella pneumoniae*
GCF_011037595.1_ASM1103759v1_genomic	GCF_011037595.1	Brazil	0_0_105_0_0_0_1_11_0_0	*Klebsiella pneumoniae*
GCF_011037615.1_ASM1103761v1_genomic	GCF_011037615.1	Brazil	0_0_105_0_0_0_1_1_1_0	*Klebsiella pneumoniae*
GCF_011037635.1_ASM1103763v1_genomic	GCF_011037635.1	Brazil	0_0_105_0_0_0_1_12_0_0	*Klebsiella pneumoniae*
GCF_011037655.1_ASM1103765v1_genomic	GCF_011037655.1	Brazil	0_0_105_0_0_0_1_7_0_0	*Klebsiella pneumoniae*
GCF_011037675.1_ASM1103767v1_genomic	GCF_011037675.1	Brazil	0_0_105_0_0_0_13_1_0_0	*Klebsiella pneumoniae*
GCF_011037685.1_ASM1103768v1_genomic	GCF_011037685.1	Brazil	0_0_105_0_9_0_19_0_0_0	*Klebsiella pneumoniae*
GCF_011037715.1_ASM1103771v1_genomic	GCF_011037715.1	Brazil	0_0_105_0_0_0_1_4_0_0	*Klebsiella pneumoniae*
GCF_011037755.1_ASM1103775v1_genomic	GCF_011037755.1	Brazil	0_0_105_0_0_0_0_1_1_0	*Klebsiella pneumoniae*
GCF_011037775.1_ASM1103777v1_genomic	GCF_011037775.1	Brazil	0_0_105_0_0_0_0_1_2_0	*Klebsiella pneumoniae*
GCF_011037865.1_ASM1103786v1_genomic	GCF_011037865.1	Brazil	0_0_105_6_0_0_69_0_0_0	*Klebsiella pneumoniae*
GCF_011037885.1_ASM1103788v1_genomic	GCF_011037885.1	Brazil	0_0_105_6_0_0_69_0_0_0	*Klebsiella pneumoniae*
GCF_011037925.1_ASM1103792v1_genomic	GCF_011037925.1	Brazil	0_0_105_6_0_0_69_0_0_0	*Klebsiella pneumoniae*
GCF_011037945.1_ASM1103794v1_genomic	GCF_011037945.1	Brazil	0_0_105_6_0_0_69_0_6_0	*Klebsiella pneumoniae*
GCF_011037955.1_ASM1103795v1_genomic	GCF_011037955.1	Brazil	0_0_105_6_0_0_69_0_8_0	*Klebsiella pneumoniae*
GCF_011037995.1_ASM1103799v1_genomic	GCF_011037995.1	Brazil	0_0_105_6_0_0_69_0_0_0	*Klebsiella pneumoniae*

GCF_011038015.1_ASM1103801v1_genomic	GCF_011038015.1	Brazil	0_0_105_6_0_0_69_0_9_0	*Klebsiella pneumoniae*
GCF_011038035.1_ASM1103803v1_genomic	GCF_011038035.1	Brazil	0_0_105_6_0_0_69_0_0_0	*Klebsiella pneumoniae*
GCF_011038055.1_ASM1103805v1_genomic	GCF_011038055.1	Brazil	0_0_105_6_0_0_69_0_0_0	*Klebsiella pneumoniae*
GCF_011038095.1_ASM1103809v1_genomic	GCF_011038095.1	Brazil	0_0_105_6_0_0_69_0_10_0	*Klebsiella pneumoniae*
GCF_011038115.1_ASM1103811v1_genomic	GCF_011038115.1	Brazil	0_0_105_6_0_0_69_0_11_0	*Klebsiella pneumoniae*
GCF_011038135.1_ASM1103813v1_genomic	GCF_011038135.1	Brazil	0_0_105_6_0_0_69_0_0_0	*Klebsiella pneumoniae*
GCF_011038155.1_ASM1103815v1_genomic	GCF_011038155.1	Brazil	0_0_22_27_0_0_0_120_0_0	*Klebsiella pneumoniae*
GCF_011038175.1_ASM1103817v1_genomic	GCF_011038175.1	Brazil	0_0_22_27_0_0_0_0_27_0	*Klebsiella pneumoniae*
GCF_011038195.1_ASM1103819v1_genomic	GCF_011038195.1	Brazil	0_0_22_27_0_0_0_0_28_0	*Klebsiella pneumoniae*
GCF_011038215.1_ASM1103821v1_genomic	GCF_011038215.1	Brazil	0_0_22_27_0_0_84_0_0_0	*Klebsiella pneumoniae*
GCF_011038235.1_ASM1103823v1_genomic	GCF_011038235.1	Brazil	0_0_22_27_0_0_0_0_29_0	*Klebsiella pneumoniae*
GCF_011038255.1_ASM1103825v1_genomic	GCF_011038255.1	Brazil	0_0_22_27_0_0_0_0_30_0	*Klebsiella pneumoniae*
GCF_011038265.1_ASM1103826v1_genomic	GCF_011038265.1	Brazil	0_0_22_27_0_0_0_121_0_0	*Klebsiella pneumoniae*
GCF_011038295.1_ASM1103829v1_genomic	GCF_011038295.1	Brazil	0_0_22_27_0_0_0_0_31_0	*Klebsiella pneumoniae*
GCF_011038315.1_ASM1103831v1_genomic	GCF_011038315.1	Brazil	0_0_22_27_0_9_1_0_0_0	*Klebsiella pneumoniae*
GCF_011038335.1_ASM1103833v1_genomic	GCF_011038335.1	Brazil	0_0_22_27_0_0_0_0_32_0	*Klebsiella pneumoniae*
GCF_011038355.1_ASM1103835v1_genomic	GCF_011038355.1	Brazil	0_0_22_27_0_0_0_122_0_0	*Klebsiella pneumoniae*
GCF_011038365.1_ASM1103836v1_genomic	GCF_011038365.1	Brazil	0_0_22_27_0_0_0_0_33_0	*Klebsiella pneumoniae*
GCF_011038395.1_ASM1103839v1_genomic	GCF_011038395.1	Brazil	0_0_22_27_0_0_0_0_34_0	*Klebsiella pneumoniae*
GCF_011038415.1_ASM1103841v1_genomic	GCF_011038415.1	Brazil	0_0_22_27_0_0_0_0_35_0	*Klebsiella pneumoniae*
GCF_011038425.1_ASM1103842v1_genomic	GCF_011038425.1	Brazil	0_0_22_27_0_0_0_0_36_0	*Klebsiella pneumoniae*
GCF_011038455.1_ASM1103845v1_genomic	GCF_011038455.1	Brazil	0_0_22_27_0_0_0_0_37_0	*Klebsiella pneumoniae*
GCF_011038475.1_ASM1103847v1_genomic	GCF_011038475.1	Brazil	0_0_22_27_0_0_0_0_38_0	*Klebsiella pneumoniae*
GCF_011038495.1_ASM1103849v1_genomic	GCF_011038495.1	Brazil	0_0_22_27_0_0_0_123_0_0	*Klebsiella pneumoniae*
GCF_011038515.1_ASM1103851v1_genomic	GCF_011038515.1	Brazil	0_0_22_27_0_0_0_0_39_0	*Klebsiella pneumoniae*
GCF_011601045.1_ASM1160104v1_genomic	GCF_011601045.1	Brazil	0_0_105_6_0_0_69_0_0_0	*Klebsiella pneumoniae*
GCF_011684055.1_ASM1168405v1_genomic	GCF_011684055.1	Brazil	0_0_1302_0_0_0_0_0_0_0	*Klebsiella pneumoniae*
GCF_012102235.1_ASM1210223v1_genomic	GCF_012102235.1	Brazil	0_0_105_6_0_0_69_0_0_0	*Klebsiella pneumoniae*
GCF_012102535.1_ASM1210253v1_genomic	GCF_012102535.1	Brazil	0_0_322_0_1_0_1_0_0_0	*Klebsiella pneumoniae*
GCF_012241505.1_ASM1224150v1_genomic	GCF_012241505.1	Peru	0_0_197_0_4_30_0_1_0_0	*Klebsiella pneumoniae*
GCF_012241595.1_ASM1224159v1_genomic	GCF_012241595.1	Brazil	0_0_0_0_2_0_0_31_0_0	*Klebsiella pneumoniae*
GCF_012955865.1_ASM1295586v1_genomic	GCF_012955865.1	Brazil	0_0_105_0_0_11_0_0_0_0	*Klebsiella pneumoniae*
GCF_012955875.1_ASM1295587v1_genomic	GCF_012955875.1	Brazil	0_0_105_0_0_0_25_0_0_0	*Klebsiella pneumoniae*
GCF_012955905.1_ASM1295590v1_genomic	GCF_012955905.1	Brazil	0_0_105_0_9_0_0_28_1_0	*Klebsiella pneumoniae*

GCF_012955925.1_ASM1295592v1_genomic	GCF_012955925.1	Brazil	0_0_105_0_76_0_0_0_0_0	*Klebsiella pneumoniae*
GCF_012956605.1_ASM1295660v1_genomic	GCF_012956605.1	Brazil	0_0_105_6_0_0_379_0_0_0	*Klebsiella pneumoniae*
GCF_012956625.1_ASM1295662v1_genomic	GCF_012956625.1	Brazil	0_0_105_6_0_0_69_3_0_0	*Klebsiella pneumoniae*
GCF_012956645.1_ASM1295664v1_genomic	GCF_012956645.1	Brazil	0_0_105_6_0_0_380_0_0_0	*Klebsiella pneumoniae*
GCF_013002785.1_ASM1300278v1_genomic	GCF_013002785.1	Brazil	0_0_105_0_24_0_0_0_3_0	*Klebsiella pneumoniae*
GCF_013155005.1_ASM1315500v1_genomic	GCF_013155005.1	Chile	0_0_137_8_0_0_0_2_0_0	*Klebsiella pneumoniae*
GCF_013169065.1_ASM1316906v1_genomic	GCF_013169065.1	Colombia	0_0_283_0_0_0_1_0_0_0	*Klebsiella pneumoniae*
GCF_013169115.1_ASM1316911v1_genomic	GCF_013169115.1	Colombia	0_0_22_24_7_1_2_0_0_0	*Klebsiella pneumoniae*
GCF_013169725.1_ASM1316972v1_genomic	GCF_013169725.1	Colombia	0_0_22_25_10_0_0_0_0_0	*Klebsiella pneumoniae*
GCF_013169765.1_ASM1316976v1_genomic	GCF_013169765.1	Colombia	0_0_105_6_0_0_17_53_75_0	*Klebsiella pneumoniae*
GCF_013169775.1_ASM1316977v1_genomic	GCF_013169775.1	Colombia	0_0_105_6_0_0_17_16_0_0	*Klebsiella pneumoniae*
GCF_013169865.1_ASM1316986v1_genomic	GCF_013169865.1	Colombia	0_0_219_0_0_0_0_1_0_0	*Klebsiella pneumoniae*
GCF_013169875.1_ASM1316987v1_genomic	GCF_013169875.1	Colombia	0_0_22_24_7_1_0_1_0_0	*Klebsiella pneumoniae*
GCF_013170105.1_ASM1317010v1_genomic	GCF_013170105.1	Colombia	0_0_29_5_0_0_0_0_2_0	*Klebsiella pneumoniae*
GCF_013170115.1_ASM1317011v1_genomic	GCF_013170115.1	Colombia	0_0_105_6_0_0_17_53_65_0	*Klebsiella pneumoniae*
GCF_013170165.1_ASM1317016v1_genomic	GCF_013170165.1	Colombia	0_0_0_0_23_1_6_0_0_0	*Klebsiella pneumoniae*
GCF_013170205.1_ASM1317020v1_genomic	GCF_013170205.1	Colombia	0_0_105_6_0_0_17_53_76_0	*Klebsiella pneumoniae*
GCF_013170215.1_ASM1317021v1_genomic	GCF_013170215.1	Colombia	0_0_388_0_5_0_1_0_0_0	*Klebsiella pneumoniae*
GCF_013170325.1_ASM1317032v1_genomic	GCF_013170325.1	Colombia	0_0_28_0_0_0_0_1_0_0	*Klebsiella pneumoniae*
GCF_013170385.1_ASM1317038v1_genomic	GCF_013170385.1	Colombia	0_0_219_0_0_0_0_2_0_0	*Klebsiella pneumoniae*
GCF_013170405.1_ASM1317040v1_genomic	GCF_013170405.1	Colombia	0_0_219_0_0_0_0_3_0_0	*Klebsiella pneumoniae*
GCF_013170425.1_ASM1317042v1_genomic	GCF_013170425.1	Colombia	0_0_472_0_4_0_0_1_0_0	*Klebsiella pneumoniae*
GCF_013170435.1_ASM1317043v1_genomic	GCF_013170435.1	Colombia	0_0_84_0_0_6_0_0_0_0	*Klebsiella pneumoniae*
GCF_013170445.1_ASM1317044v1_genomic	GCF_013170445.1	Colombia	0_0_219_0_0_0_0_4_0_0	*Klebsiella pneumoniae*
GCF_013170455.1_ASM1317045v1_genomic	GCF_013170455.1	Colombia	0_0_219_0_0_0_0_5_0_0	*Klebsiella pneumoniae*
GCF_013170505.1_ASM1317050v1_genomic	GCF_013170505.1	Colombia	0_0_472_0_4_0_0_0_1_0	*Klebsiella pneumoniae*
GCF_013170535.1_ASM1317053v1_genomic	GCF_013170535.1	Colombia	0_0_219_0_0_0_0_6_0_0	*Klebsiella pneumoniae*
GCF_013170565.1_ASM1317056v1_genomic	GCF_013170565.1	Colombia	0_0_219_0_0_0_0_7_0_0	*Klebsiella pneumoniae*
GCF_013170595.1_ASM1317059v1_genomic	GCF_013170595.1	Colombia	0_0_219_0_0_0_0_8_0_0	*Klebsiella pneumoniae*
GCF_013170625.1_ASM1317062v1_genomic	GCF_013170625.1	Colombia	0_0_219_0_0_0_0_9_0_0	*Klebsiella pneumoniae*
GCF_013170645.1_ASM1317064v1_genomic	GCF_013170645.1	Colombia	0_0_219_0_0_0_0_10_0_0	*Klebsiella pneumoniae*
GCF_013303005.1_ASM1330300v1_genomic	GCF_013303005.1	Brazil	0_0_105_0_9_0_0_28_0_0	*Klebsiella pneumoniae*
GCF_013372785.1_ASM1337278v1_genomic	GCF_013372785.1	Brazil	0_0_22_27_0_0_0_0_0_0	*Klebsiella pneumoniae*
GCF_014323585.1_ASM1432358v1_genomic	GCF_014323585.1	Brazil	0_0_105_1_1_1_0_0_0_0	*Klebsiella pneumoniae*

GCF_014323625.1_ASM1432362v1_genomic	GCF_014323625.1	Brazil	0_0_105_6_0_0_17_182_0_0	*Klebsiella pneumoniae*
GCF_014323715.1_ASM1432371v1_genomic	GCF_014323715.1	Brazil	0_0_105_0_0_0_1_13_0_0	*Klebsiella pneumoniae*
GCF_014451135.1_ASM1445113v1_genomic	GCF_014451135.1	Brazil	0_0_220_0_0_0_11_0_0_0	*Klebsiella pneumoniae*
GCF_014451175.1_ASM1445117v1_genomic	GCF_014451175.1	Brazil	0_0_220_0_0_0_12_0_0_0	*Klebsiella pneumoniae*
GCF_014451185.1_ASM1445118v1_genomic	GCF_014451185.1	Brazil	0_0_220_0_8_0_0_0_0_0	*Klebsiella pneumoniae*
GCF_014451215.1_ASM1445121v1_genomic	GCF_014451215.1	Brazil	0_0_0_0_80_0_0_0_0_0	*Klebsiella pneumoniae*
GCF_014451235.1_ASM1445123v1_genomic	GCF_014451235.1	Brazil	0_0_105_1_1_1_4_3_0_0	*Klebsiella pneumoniae*
GCF_014451245.1_ASM1445124v1_genomic	GCF_014451245.1	Brazil	0_0_105_6_0_0_69_0_0_0	*Klebsiella pneumoniae*
GCF_014451255.1_ASM1445125v1_genomic	GCF_014451255.1	Brazil	0_0_105_1_1_1_3_1_0_0	*Klebsiella pneumoniae*
GCF_014451275.1_ASM1445127v1_genomic	GCF_014451275.1	Brazil	0_0_0_0_2_0_165_0_0_0	*Klebsiella pneumoniae*
GCF_014451315.1_ASM1445131v1_genomic	GCF_014451315.1	Brazil	0_0_105_6_0_0_69_4_0_0	*Klebsiella pneumoniae*
GCF_014490685.2_ASM1449068v2_genomic	GCF_014490685.2	Peru	0_0_197_0_4_30_1_0_0_0	*Klebsiella pneumoniae*
GCF_014500765.1_ASM1450076v1_genomic	GCF_014500765.1	Brazil	0_0_0_0_2_0_0_0_67_0	*Klebsiella pneumoniae*
GCF_014500785.1_ASM1450078v1_genomic	GCF_014500785.1	Brazil	0_0_0_0_2_0_0_227_0_0	*Klebsiella pneumoniae*
GCF_014500805.1_ASM1450080v1_genomic	GCF_014500805.1	Brazil	0_0_0_0_2_0_0_0_68_0	*Klebsiella pneumoniae*
GCF_014500815.1_ASM1450081v1_genomic	GCF_014500815.1	Brazil	0_0_0_0_2_0_0_0_69_0	*Klebsiella pneumoniae*
GCF_014500865.1_ASM1450086v1_genomic	GCF_014500865.1	Brazil	0_0_0_0_2_0_125_1_0_0	*Klebsiella pneumoniae*
GCF_014524525.1_ASM1452452v1_genomic	GCF_014524525.1	Brazil	0_0_0_0_2_0_0_0_70_0	*Klebsiella pneumoniae*
GCF_014788765.1_ASM1478876v1_genomic	GCF_014788765.1	Chile	0_0_388_0_1_0_9_2_0_0	*Klebsiella pneumoniae*
GCF_014833135.1_Kp14U04_genomic	GCF_014833135.1	Brazil	0_0_0_0_2_0_166_0_0_0	*Klebsiella pneumoniae*
GCF_014883955.1_ASM1488395v1_genomic	GCF_014883955.1	Colombia	0_0_128_9_2_0_0_0_0_0	*Klebsiella pneumoniae*
GCF_015666295.1_ASM1566629v1_genomic	GCF_015666295.1	Brazil	0_0_105_0_3_0_1_13_0_0	*Klebsiella pneumoniae*
GCF_015666325.1_ASM1566632v1_genomic	GCF_015666325.1	Brazil	0_0_105_0_0_0_13_0_0_0	*Klebsiella pneumoniae*
GCF_015666395.1_ASM1566639v1_genomic	GCF_015666395.1	Brazil	0_0_0_0_37_1_0_1_0_0	*Klebsiella pneumoniae*
GCF_015666405.1_ASM1566640v1_genomic	GCF_015666405.1	Brazil	0_0_105_0_3_0_28_2_0_0	*Klebsiella pneumoniae*
GCF_015666415.1_ASM1566641v1_genomic	GCF_015666415.1	Brazil	0_0_105_0_0_0_13_0_0_0	*Klebsiella pneumoniae*
GCF_015666445.1_ASM1566644v1_genomic	GCF_015666445.1	Brazil	0_0_105_0_0_0_13_0_0_0	*Klebsiella pneumoniae*
GCF_015666485.1_ASM1566648v1_genomic	GCF_015666485.1	Brazil	0_0_105_0_3_0_63_0_0_0	*Klebsiella pneumoniae*
GCF_015666525.1_ASM1566652v1_genomic	GCF_015666525.1	Brazil	0_0_124_0_0_2_0_0_0_0	*Klebsiella pneumoniae*
GCF_015666585.1_ASM1566658v1_genomic	GCF_015666585.1	Brazil	0_0_105_0_3_0_28_3_0_0	*Klebsiella pneumoniae*
GCF_015666595.1_ASM1566659v1_genomic	GCF_015666595.1	Brazil	0_0_105_6_0_0_69_0_0_0	*Klebsiella pneumoniae*
GCF_015666615.1_ASM1566661v1_genomic	GCF_015666615.1	Brazil	0_0_369_0_0_0_0_380_0_0	*Klebsiella pneumoniae*
GCF_015666635.1_ASM1566663v1_genomic	GCF_015666635.1	Brazil	0_0_194_0_0_0_2_0_0_0	*Klebsiella pneumoniae*
GCF_015666685.1_ASM1566668v1_genomic	GCF_015666685.1	Brazil	0_0_419_0_0_0_2_0_0_0	*Klebsiella pneumoniae*

GCF_015826235.1_ASM1582623v1_genomic	GCF_015826235.1	Brazil	0_0_395_0_59_0_0_0_0_0	*Klebsiella pneumoniae*
GCF_015826265.1_ASM1582626v1_genomic	GCF_015826265.1	Brazil	0_0_395_0_60_0_0_0_0_0	*Klebsiella pneumoniae*
GCF_015912475.1_ASM1591247v1_genomic	GCF_015912475.1	Uruguay	0_0_105_6_0_0_381_0_0_0	*Klebsiella pneumoniae*
GCF_015912485.1_ASM1591248v1_genomic	GCF_015912485.1	Uruguay	0_0_105_6_0_0_382_0_0_0	*Klebsiella pneumoniae*
GCF_015912585.1_ASM1591258v1_genomic	GCF_015912585.1	Uruguay	0_0_105_6_0_0_383_0_0_0	*Klebsiella pneumoniae*
GCF_016054805.1_ASM1605480v1_genomic	GCF_016054805.1	Brazil	0_0_105_0_9_0_0_56_0_0	*Klebsiella pneumoniae*
GCF_016054825.1_ASM1605482v1_genomic	GCF_016054825.1	Brazil	0_0_106_0_0_2_3_0_0_0	*Klebsiella pneumoniae*
GCF_016054895.1_ASM1605489v1_genomic	GCF_016054895.1	Brazil	0_0_94_0_0_0_0_1_1_0	*Klebsiella pneumoniae*
GCF_016054935.1_ASM1605493v1_genomic	GCF_016054935.1	Brazil	0_0_124_0_0_3_0_0_0_0	*Klebsiella pneumoniae*
GCF_016054995.1_ASM1605499v1_genomic	GCF_016054995.1	Brazil	0_0_124_0_0_4_0_0_0_0	*Klebsiella pneumoniae*
GCF_016055215.1_ASM1605521v1_genomic	GCF_016055215.1	Brazil	0_0_211_0_39_0_0_0_0_0	*Klebsiella pneumoniae*
GCF_016055235.1_ASM1605523v1_genomic	GCF_016055235.1	Brazil	0_0_105_0_0_0_13_0_0_0	*Klebsiella pneumoniae*
GCF_016055275.1_ASM1605527v1_genomic	GCF_016055275.1	Brazil	0_0_105_0_0_0_1_14_0_0	*Klebsiella pneumoniae*
GCF_016055295.1_ASM1605529v1_genomic	GCF_016055295.1	Brazil	0_0_194_0_0_0_3_0_0_0	*Klebsiella pneumoniae*
GCF_016055305.1_ASM1605530v1_genomic	GCF_016055305.1	Brazil	0_0_323_0_1_0_1_1_0_0	*Klebsiella pneumoniae*
GCF_016055345.1_ASM1605534v1_genomic	GCF_016055345.1	Brazil	0_0_197_0_25_0_0_23_0_0	*Klebsiella pneumoniae*
GCF_016055395.1_ASM1605539v1_genomic	GCF_016055395.1	Brazil	0_0_105_0_3_0_28_1_0_0	*Klebsiella pneumoniae*
GCF_016055435.1_ASM1605543v1_genomic	GCF_016055435.1	Brazil	0_0_369_0_0_0_0_0_1_0	*Klebsiella pneumoniae*
GCF_016055445.1_ASM1605544v1_genomic	GCF_016055445.1	Brazil	0_0_197_0_25_0_0_23_0_0	*Klebsiella pneumoniae*
GCF_016055495.1_ASM1605549v1_genomic	GCF_016055495.1	Brazil	0_0_105_6_0_0_69_0_0_0	*Klebsiella pneumoniae*
GCF_016055535.1_ASM1605553v1_genomic	GCF_016055535.1	Brazil	0_0_105_6_0_0_69_0_12_0	*Klebsiella pneumoniae*
GCF_016055575.1_ASM1605557v1_genomic	GCF_016055575.1	Brazil	0_0_22_27_0_0_0_106_3_0	*Klebsiella pneumoniae*
GCF_016055605.1_ASM1605560v1_genomic	GCF_016055605.1	Brazil	0_0_197_0_4_0_1_3_4_0	*Klebsiella pneumoniae*
GCF_016055635.1_ASM1605563v1_genomic	GCF_016055635.1	Brazil	0_0_197_0_0_0_20_0_0_0	*Klebsiella pneumoniae*
GCF_016055655.1_ASM1605565v1_genomic	GCF_016055655.1	Brazil	0_0_22_24_7_1_3_0_0_0	*Klebsiella pneumoniae*
GCF_016055675.1_ASM1605567v1_genomic	GCF_016055675.1	Brazil	0_0_105_6_0_0_69_0_0_0	*Klebsiella pneumoniae*
GCF_016055695.1_ASM1605569v1_genomic	GCF_016055695.1	Brazil	0_0_105_0_0_0_13_0_0_0	*Klebsiella pneumoniae*
GCF_016055755.1_ASM1605575v1_genomic	GCF_016055755.1	Brazil	0_0_105_0_0_0_1_15_0_0	*Klebsiella pneumoniae*
GCF_016055795.1_ASM1605579v1_genomic	GCF_016055795.1	Brazil	0_0_323_0_1_0_1_2_0_0	*Klebsiella pneumoniae*
GCF_016055885.1_ASM1605588v1_genomic	GCF_016055885.1	Brazil	0_0_105_0_0_0_1_16_0_0	*Klebsiella pneumoniae*
GCF_016055895.1_ASM1605589v1_genomic	GCF_016055895.1	Brazil	0_0_105_0_3_0_28_1_0_0	*Klebsiella pneumoniae*
GCF_016055935.1_ASM1605593v1_genomic	GCF_016055935.1	Brazil	0_0_84_0_8_0_4_8_0_0	*Klebsiella pneumoniae*
GCF_016055975.1_ASM1605597v1_genomic	GCF_016055975.1	Brazil	0_0_107_0_4_0_1_9_0_0	*Klebsiella pneumoniae*
GCF_016055995.1_ASM1605599v1_genomic	GCF_016055995.1	Brazil	0_0_105_0_0_0_1_17_0_0	*Klebsiella pneumoniae*

GCF_016056105.1_ASM1605610v1_genomic	GCF_016056105.1	Brazil	0_0_105_1_1_1_3_2_0_0	*Klebsiella pneumoniae*
GCF_016056175.1_ASM1605617v1_genomic	GCF_016056175.1	Brazil	0_0_369_0_0_0_0_0_104_0	*Klebsiella pneumoniae*
GCF_016056195.1_ASM1605619v1_genomic	GCF_016056195.1	Brazil	0_0_0_0_2_0_0_0_72_0	*Klebsiella pneumoniae*
GCF_016801375.1_ASM1680137v1_genomic	GCF_016801375.1	Colombia	0_0_105_6_0_0_17_722_0_0	*Klebsiella pneumoniae*
GCF_016805425.1_ASM1680542v1_genomic	GCF_016805425.1	Ecuador	0_0_98_0_5_0_0_2_0_0	*Klebsiella pneumoniae*
GCF_016805435.1_ASM1680543v1_genomic	GCF_016805435.1	Ecuador	0_0_98_0_5_0_0_3_0_0	*Klebsiella pneumoniae*
GCF_016805465.1_ASM1680546v1_genomic	GCF_016805465.1	Ecuador	0_0_98_0_5_0_0_0_7_0	*Klebsiella pneumoniae*
GCF_016805485.1_ASM1680548v1_genomic	GCF_016805485.1	Ecuador	0_0_98_0_5_0_0_4_0_0	*Klebsiella pneumoniae*
GCF_016805505.1_ASM1680550v1_genomic	GCF_016805505.1	Ecuador	0_0_98_0_5_0_0_5_0_0	*Klebsiella pneumoniae*
GCF_016805525.1_ASM1680552v1_genomic	GCF_016805525.1	Ecuador	0_0_98_0_5_0_0_0_8_0	*Klebsiella pneumoniae*
GCF_016805545.1_ASM1680554v1_genomic	GCF_016805545.1	Ecuador	0_0_98_0_5_0_0_6_0_0	*Klebsiella pneumoniae*
GCF_016805565.1_ASM1680556v1_genomic	GCF_016805565.1	Ecuador	0_0_105_6_43_0_0_0_1_0	*Klebsiella pneumoniae*
GCF_016805585.1_ASM1680558v1_genomic	GCF_016805585.1	Ecuador	0_0_105_6_43_0_2_0_0_0	*Klebsiella pneumoniae*
GCF_016805625.1_ASM1680562v1_genomic	GCF_016805625.1	Ecuador	0_0_105_6_43_0_0_4_0_0	*Klebsiella pneumoniae*
GCF_016805635.1_ASM1680563v1_genomic	GCF_016805635.1	Ecuador	0_0_98_0_5_0_1_0_0_0	*Klebsiella pneumoniae*
GCF_016805665.1_ASM1680566v1_genomic	GCF_016805665.1	Ecuador	0_0_98_0_5_0_0_0_9_0	*Klebsiella pneumoniae*
GCF_016805685.1_ASM1680568v1_genomic	GCF_016805685.1	Ecuador	0_0_98_0_5_0_0_0_10_0	*Klebsiella pneumoniae*
GCF_016805705.1_ASM1680570v1_genomic	GCF_016805705.1	Ecuador	0_0_98_0_5_0_2_0_0_0	*Klebsiella pneumoniae*
GCF_016805715.1_ASM1680571v1_genomic	GCF_016805715.1	Ecuador	0_0_98_0_5_0_0_0_11_0	*Klebsiella pneumoniae*
GCF_016805745.1_ASM1680574v1_genomic	GCF_016805745.1	Ecuador	0_0_395_0_12_0_4_0_0_0	*Klebsiella pneumoniae*
GCF_016805765.1_ASM1680576v1_genomic	GCF_016805765.1	Ecuador	0_0_105_6_43_0_0_1_1_0	*Klebsiella pneumoniae*
GCF_016805785.1_ASM1680578v1_genomic	GCF_016805785.1	Ecuador	0_0_105_6_43_0_0_5_0_0	*Klebsiella pneumoniae*
GCF_016836885.1_ASM1683688v1_genomic	GCF_016836885.1	Paraguay	0_0_848_0_0_0_0_0_0_0	*Klebsiella pneumoniae*
GCF_016836905.1_ASM1683690v1_genomic	GCF_016836905.1	Paraguay	0_0_261_0_0_0_0_8_0_0	*Klebsiella pneumoniae*
GCF_016836965.1_ASM1683696v1_genomic	GCF_016836965.1	Paraguay	0_0_0_0_2_58_0_0_0_0	*Klebsiella pneumoniae*
GCF_016837025.1_ASM1683702v1_genomic	GCF_016837025.1	Paraguay	0_0_158_7_1_2_1_0_0_0	*Klebsiella pneumoniae*
GCF_016837045.1_ASM1683704v1_genomic	GCF_016837045.1	Paraguay	0_0_123_1_5_0_0_0_0_0	*Klebsiella pneumoniae*
GCF_016887985.1_ASM1688798v1_genomic	GCF_016887985.1	Paraguay	0_0_158_7_1_2_2_0_0_0	*Klebsiella pneumoniae*
GCF_017309465.1_ASM1730946v1_genomic	GCF_017309465.1	Paraguay	0_0_88_0_0_0_2_5_1_0	*Klebsiella pneumoniae*
GCF_017655225.1_ASM1765522v1_genomic	GCF_017655225.1	Peru	0_0_369_0_0_0_0_0_100_0	*Klebsiella pneumoniae*
GCF_018109905.1_ASM1810990v1_genomic	GCF_018109905.1	Brazil	0_0_0_0_2_0_75_2_0_0	*Klebsiella pneumoniae*
GCF_018359185.1_ASM1835918v1_genomic	GCF_018359185.1	Colombia	0_0_95_1_3_0_2_0_0_0	*Klebsiella pneumoniae*
GCF_018359195.1_ASM1835919v1_genomic	GCF_018359195.1	Colombia	0_0_105_6_0_0_17_689_1_0	*Klebsiella pneumoniae*
GCF_018359205.1_ASM1835920v1_genomic	GCF_018359205.1	Colombia	0_0_105_6_0_0_17_689_1_0	*Klebsiella pneumoniae*

GCF_018359245.1_ASM1835924v1_genomic	GCF_018359245.1	Colombia	0_0_95_1_3_0_3_0_0_0	*Klebsiella pneumoniae*
GCF_018359255.1_ASM1835925v1_genomic	GCF_018359255.1	Colombia	0_0_105_6_0_0_17_689_1_0	*Klebsiella pneumoniae*
GCF_018359285.1_ASM1835928v1_genomic	GCF_018359285.1	Colombia	0_0_105_6_0_0_17_16_6_0	*Klebsiella pneumoniae*
GCF_018359295.1_ASM1835929v1_genomic	GCF_018359295.1	Colombia	0_0_80_8_0_0_0_0_0_0	*Klebsiella pneumoniae*
GCF_018359305.1_ASM1835930v1_genomic	GCF_018359305.1	Colombia	0_0_105_6_43_0_0_1_0_0	*Klebsiella pneumoniae*
GCF_018359345.1_ASM1835934v1_genomic	GCF_018359345.1	Colombia	0_0_105_6_0_0_17_16_0_0	*Klebsiella pneumoniae*
GCF_018359365.1_ASM1835936v1_genomic	GCF_018359365.1	Colombia	0_0_105_6_0_0_17_697_0_0	*Klebsiella pneumoniae*
GCF_018359385.1_ASM1835938v1_genomic	GCF_018359385.1	Colombia	0_0_105_6_0_0_17_16_6_0	*Klebsiella pneumoniae*
GCF_018359405.1_ASM1835940v1_genomic	GCF_018359405.1	Colombia	0_0_105_6_0_0_17_16_6_0	*Klebsiella pneumoniae*
GCF_018359425.1_ASM1835942v1_genomic	GCF_018359425.1	Colombia	0_0_105_6_0_0_17_16_6_0	*Klebsiella pneumoniae*
GCF_018359445.1_ASM1835944v1_genomic	GCF_018359445.1	Colombia	0_0_105_6_0_0_17_16_0_0	*Klebsiella pneumoniae*
GCF_018359455.1_ASM1835945v1_genomic	GCF_018359455.1	Colombia	0_0_105_6_0_0_17_16_35_0	*Klebsiella pneumoniae*
GCF_018359485.1_ASM1835948v1_genomic	GCF_018359485.1	Colombia	0_0_1_1_0_1_1_0_0_6	*Klebsiella pneumoniae*
GCF_018359505.1_ASM1835950v1_genomic	GCF_018359505.1	Colombia	0_0_1_1_0_1_1_0_0_7	*Klebsiella pneumoniae*
GCF_018359525.1_ASM1835952v1_genomic	GCF_018359525.1	Colombia	0_0_1_1_0_1_1_0_5_0	*Klebsiella pneumoniae*
GCF_018359545.1_ASM1835954v1_genomic	GCF_018359545.1	Colombia	0_0_105_6_0_0_17_16_0_0	*Klebsiella pneumoniae*
GCF_018359565.1_ASM1835956v1_genomic	GCF_018359565.1	Colombia	0_0_1_1_0_1_1_0_0_8	*Klebsiella pneumoniae*
GCF_018359585.1_ASM1835958v1_genomic	GCF_018359585.1	Colombia	0_0_105_6_0_0_17_16_0_0	*Klebsiella pneumoniae*
GCF_018359605.1_ASM1835960v1_genomic	GCF_018359605.1	Colombia	0_0_105_6_0_0_17_16_0_0	*Klebsiella pneumoniae*
GCF_018359625.1_ASM1835962v1_genomic	GCF_018359625.1	Colombia	0_0_105_6_0_0_17_16_0_0	*Klebsiella pneumoniae*
GCF_018359645.1_ASM1835964v1_genomic	GCF_018359645.1	Colombia	0_0_105_6_0_0_17_53_1_0	*Klebsiella pneumoniae*
GCF_018423625.1_ASM1842362v1_genomic	GCF_018423625.1	Brazil	0_0_105_0_0_0_1_18_0_0	*Klebsiella pneumoniae*
GCF_018423645.1_ASM1842364v1_genomic	GCF_018423645.1	Brazil	0_0_105_1_1_1_19_1_0_0	*Klebsiella pneumoniae*
GCF_018423665.1_ASM1842366v1_genomic	GCF_018423665.1	Brazil	0_0_105_0_0_0_1_19_0_0	*Klebsiella pneumoniae*
GCF_018423675.1_ASM1842367v1_genomic	GCF_018423675.1	Brazil	0_0_105_0_24_0_0_5_1_0	*Klebsiella pneumoniae*
GCF_018423705.1_ASM1842370v1_genomic	GCF_018423705.1	Brazil	0_0_105_1_1_1_0_0_0_0	*Klebsiella pneumoniae*
GCF_018423715.1_ASM1842371v1_genomic	GCF_018423715.1	Brazil	0_0_105_0_0_0_3_0_1_0	*Klebsiella pneumoniae*
GCF_018423745.1_ASM1842374v1_genomic	GCF_018423745.1	Brazil	0_0_105_0_0_0_1_1_1_0	*Klebsiella pneumoniae*
GCF_018423765.1_ASM1842376v1_genomic	GCF_018423765.1	Brazil	0_0_105_0_24_0_0_0_4_0	*Klebsiella pneumoniae*
GCF_018423775.1_ASM1842377v1_genomic	GCF_018423775.1	Brazil	0_0_105_1_1_1_0_0_0_0	*Klebsiella pneumoniae*
GCF_018423805.1_ASM1842380v1_genomic	GCF_018423805.1	Brazil	0_0_105_0_0_0_1_20_0_0	*Klebsiella pneumoniae*
GCF_018423815.1_ASM1842381v1_genomic	GCF_018423815.1	Brazil	0_0_105_1_1_1_0_0_0_0	*Klebsiella pneumoniae*
GCF_018423825.1_ASM1842382v1_genomic	GCF_018423825.1	Brazil	0_0_22_27_0_0_0_0_41_0	*Klebsiella pneumoniae*
GCF_018423865.1_ASM1842386v1_genomic	GCF_018423865.1	Brazil	0_0_105_1_1_5_0_2_0_0	*Klebsiella pneumoniae*

GCF_018423905.1_ASM1842390v1_genomic	GCF_018423905.1	Brazil	0_0_105_1_1_1_0_0_4_0	*Klebsiella pneumoniae*
GCF_018423915.1_ASM1842391v1_genomic	GCF_018423915.1	Brazil	0_0_105_1_1_1_4_0_1_0	*Klebsiella pneumoniae*
GCF_018920475.1_ASM1892047v1_genomic	GCF_018920475.1	Brazil	0_0_395_5_0_0_0_0_0_0	*Klebsiella pneumoniae*
GCF_019038575.1_ASM1903857v1_genomic	GCF_019038575.1	Brazil	0_0_883_0_0_0_0_0_0_0	*Klebsiella pneumoniae*
GCF_019334205.1_ASM1933420v1_genomic	GCF_019334205.1	Brazil	0_0_219_4_0_0_1_0_0_0	*Klebsiella pneumoniae*
GCF_019837225.1_ASM1983722v1_genomic	GCF_019837225.1	Brazil	0_0_105_0_85_0_0_0_0_0	*Klebsiella pneumoniae*
GCF_019928625.2_ASM1992862v2_genomic	GCF_019928625.2	Peru	0_0_146_0_2_1_0_1_0_0	*Klebsiella pneumoniae*
GCF_019928665.1_ASM1992866v1_genomic	GCF_019928665.1	Peru	0_0_374_0_2_1_1_1_0_0	*Klebsiella pneumoniae*
GCF_020589615.1_ASM2058961v1_genomic	GCF_020589615.1	Brazil	0_0_105_0_11_0_12_0_0_0	*Klebsiella pneumoniae*
GCF_020589635.1_ASM2058963v1_genomic	GCF_020589635.1	Brazil	0_0_105_0_11_0_12_0_0_0	*Klebsiella pneumoniae*
GCF_020589645.1_ASM2058964v1_genomic	GCF_020589645.1	Brazil	0_0_194_0_0_0_4_0_0_0	*Klebsiella pneumoniae*
GCF_020589755.1_ASM2058975v1_genomic	GCF_020589755.1	Brazil	0_0_309_0_2_1_1_0_0_0	*Klebsiella pneumoniae*
GCF_020589795.1_ASM2058979v1_genomic	GCF_020589795.1	Brazil	0_0_22_12_38_0_0_0_0_0	*Klebsiella pneumoniae*
GCF_020589815.1_ASM2058981v1_genomic	GCF_020589815.1	Brazil	0_0_407_0_0_0_0_0_2_0	*Klebsiella pneumoniae*
GCF_020589835.1_ASM2058983v1_genomic	GCF_020589835.1	Brazil	0_0_407_0_0_0_0_0_0_0	*Klebsiella pneumoniae*
GCF_020589895.1_ASM2058989v1_genomic	GCF_020589895.1	Brazil	0_0_193_3_3_0_1_0_0_0	*Klebsiella pneumoniae*
GCF_020589935.1_ASM2058993v1_genomic	GCF_020589935.1	Brazil	0_0_407_0_0_0_0_0_0_0	*Klebsiella pneumoniae*
GCF_020615425.1_ASM2061542v1_genomic	GCF_020615425.1	Brazil	0_0_197_0_4_0_31_9_0_0	*Klebsiella pneumoniae*
GCF_020615495.1_ASM2061549v1_genomic	GCF_020615495.1	Brazil	0_0_105_0_0_0_6_1_0_0	*Klebsiella pneumoniae*
GCF_020615575.1_ASM2061557v1_genomic	GCF_020615575.1	Brazil	0_0_98_2_17_1_0_0_0_0	*Klebsiella pneumoniae*
GCF_020615655.1_ASM2061565v1_genomic	GCF_020615655.1	Brazil	0_0_105_0_0_0_6_2_0_0	*Klebsiella pneumoniae*
GCF_020615675.1_ASM2061567v1_genomic	GCF_020615675.1	Brazil	0_0_98_2_17_2_0_0_0_0	*Klebsiella pneumoniae*
GCF_021011315.1_ASM2101131v1_genomic	GCF_021011315.1	Argentina	0_0_105_0_11_0_0_24_0_0	*Klebsiella pneumoniae*
GCF_021020725.1_ASM2102072v1_genomic	GCF_021020725.1	Brazil	0_0_105_1_1_7_0_1_0_0	*Klebsiella pneumoniae*
GCF_021020735.1_ASM2102073v1_genomic	GCF_021020735.1	Brazil	0_0_105_0_3_0_1_13_3_0	*Klebsiella pneumoniae*
GCF_021020745.1_ASM2102074v1_genomic	GCF_021020745.1	Brazil	0_0_105_6_0_0_69_0_0_0	*Klebsiella pneumoniae*
GCF_021020815.1_ASM2102081v1_genomic	GCF_021020815.1	Brazil	0_0_105_6_0_0_69_0_13_0	*Klebsiella pneumoniae*
GCF_021020835.1_ASM2102083v1_genomic	GCF_021020835.1	Brazil	0_0_105_6_0_0_69_0_0_0	*Klebsiella pneumoniae*
GCF_021020855.1_ASM2102085v1_genomic	GCF_021020855.1	Brazil	0_0_105_6_0_0_69_0_0_0	*Klebsiella pneumoniae*
GCF_021020865.1_ASM2102086v1_genomic	GCF_021020865.1	Brazil	0_0_105_0_3_0_64_0_0_0	*Klebsiella pneumoniae*
GCF_021020875.1_ASM2102087v1_genomic	GCF_021020875.1	Brazil	0_0_105_0_9_0_0_58_0_0	*Klebsiella pneumoniae*
GCF_021020915.1_ASM2102091v1_genomic	GCF_021020915.1	Brazil	0_0_105_6_0_0_69_0_14_0	*Klebsiella pneumoniae*
GCF_021020935.1_ASM2102093v1_genomic	GCF_021020935.1	Brazil	0_0_105_6_0_0_69_0_15_0	*Klebsiella pneumoniae*
GCF_021020955.1_ASM2102095v1_genomic	GCF_021020955.1	Brazil	0_0_105_6_0_0_69_0_0_0	*Klebsiella pneumoniae*

GCF_021020985.1_ASM2102098v1_genomic	GCF_021020985.1	Brazil	0_0_105_6_0_0_69_0_0_0	*Klebsiella pneumoniae*
GCF_021021015.1_ASM2102101v1_genomic	GCF_021021015.1	Brazil	0_0_105_6_0_0_69_0_0_0	*Klebsiella pneumoniae*
GCF_021021035.1_ASM2102103v1_genomic	GCF_021021035.1	Brazil	0_0_105_6_0_0_69_0_0_0	*Klebsiella pneumoniae*
GCF_021021055.1_ASM2102105v1_genomic	GCF_021021055.1	Brazil	0_0_105_6_0_0_69_0_0_0	*Klebsiella pneumoniae*
GCF_021021075.1_ASM2102107v1_genomic	GCF_021021075.1	Brazil	0_0_105_6_0_0_69_0_0_0	*Klebsiella pneumoniae*
GCF_021021095.1_ASM2102109v1_genomic	GCF_021021095.1	Brazil	0_0_105_6_0_0_69_0_0_0	*Klebsiella pneumoniae*
GCF_021021115.1_ASM2102111v1_genomic	GCF_021021115.1	Brazil	0_0_105_6_0_0_69_0_0_0	*Klebsiella pneumoniae*
GCF_021021135.1_ASM2102113v1_genomic	GCF_021021135.1	Brazil	0_0_105_6_0_0_69_0_0_0	*Klebsiella pneumoniae*
GCF_021021155.1_ASM2102115v1_genomic	GCF_021021155.1	Brazil	0_0_105_6_0_0_69_0_16_0	*Klebsiella pneumoniae*
GCF_021021175.1_ASM2102117v1_genomic	GCF_021021175.1	Brazil	0_0_105_6_0_0_69_0_0_0	*Klebsiella pneumoniae*
GCF_021021195.1_ASM2102119v1_genomic	GCF_021021195.1	Brazil	0_0_105_0_3_0_16_0_5_0	*Klebsiella pneumoniae*
GCF_021021255.1_ASM2102125v1_genomic	GCF_021021255.1	Brazil	0_0_105_6_0_0_69_0_0_0	*Klebsiella pneumoniae*
GCF_021206795.1_ASM2120679v1_genomic	GCF_021206795.1	Brazil	0_0_22_27_0_0_0_124_0_0	*Klebsiella pneumoniae*
GCF_021245835.1_ASM2124583v1_genomic	GCF_021245835.1	Brazil	0_0_22_27_0_0_0_125_0_0	*Klebsiella pneumoniae*
GCF_021496095.1_ASM2149609v1_genomic	GCF_021496095.1	Brazil	0_0_105_0_11_0_0_0_1_0	*Klebsiella pneumoniae*
GCF_021568735.1_ASM2156873v1_genomic	GCF_021568735.1	Brazil	0_0_84_0_8_0_4_1_2_0	*Klebsiella pneumoniae*
GCF_021648785.1_ASM2164878v1_genomic	GCF_021648785.1	Brazil	0_0_0_0_2_0_0_0_39_0	*Klebsiella pneumoniae*
GCF_022359595.1_ASM2235959v1_genomic	GCF_022359595.1	Brazil	0_0_98_0_6_1_1_1_0_0	*Klebsiella pneumoniae*
GCF_022544755.1_ASM2254475v1_genomic	GCF_022544755.1	Brazil	0_0_105_0_0_0_1_0_2_0	*Klebsiella pneumoniae*
GCF_022568215.1_ASM2256821v1_genomic	GCF_022568215.1	Brazil	0_0_197_0_25_0_0_0_20_0	*Klebsiella pneumoniae*
GCF_022698265.1_ASM2269826v1_genomic	GCF_022698265.1	Argentina	0_0_388_0_5_0_2_0_0_0	*Klebsiella pneumoniae*
GCF_022848935.1_ASM2284893v1_genomic	GCF_022848935.1	Uruguay	0_0_105_6_0_0_385_0_0_0	*Klebsiella pneumoniae*
GCF_022848985.1_ASM2284898v1_genomic	GCF_022848985.1	Uruguay	0_0_105_6_0_0_386_0_0_0	*Klebsiella pneumoniae*
GCF_022848995.1_ASM2284899v1_genomic	GCF_022848995.1	Uruguay	0_0_105_6_0_0_387_0_0_0	*Klebsiella pneumoniae*
GCF_022849125.1_ASM2284912v1_genomic	GCF_022849125.1	Uruguay	0_0_105_6_0_0_388_0_0_0	*Klebsiella pneumoniae*
GCF_023059255.1_ASM2305925v1_genomic	GCF_023059255.1	Brazil	0_0_105_0_3_0_28_1_2_0	*Klebsiella pneumoniae*
GCF_023059265.1_ASM2305926v1_genomic	GCF_023059265.1	Brazil	0_0_105_6_0_0_17_0_98_0	*Klebsiella pneumoniae*
GCF_023059275.1_ASM2305927v1_genomic	GCF_023059275.1	Brazil	0_0_84_0_8_0_4_1_2_0	*Klebsiella pneumoniae*
GCF_023059285.1_ASM2305928v1_genomic	GCF_023059285.1	Brazil	0_0_105_0_3_0_1_13_0_0	*Klebsiella pneumoniae*
GCF_023059435.1_ASM2305943v1_genomic	GCF_023059435.1	Brazil	0_0_105_0_3_0_16_0_6_0	*Klebsiella pneumoniae*
GCF_023060075.1_ASM2306007v1_genomic	GCF_023060075.1	Brazil	0_0_105_6_0_0_17_731_0_0	*Klebsiella pneumoniae*
GCF_023060155.1_ASM2306015v1_genomic	GCF_023060155.1	Brazil	0_0_105_0_11_0_0_33_0_0	*Klebsiella pneumoniae*
GCF_023060175.1_ASM2306017v1_genomic	GCF_023060175.1	Brazil	0_0_22_27_0_0_0_0_44_0	*Klebsiella pneumoniae*
GCF_023060205.1_ASM2306020v1_genomic	GCF_023060205.1	Brazil	0_0_124_0_0_5_0_0_0_0	*Klebsiella pneumoniae*

GCF_023060255.1_ASM2306025v1_genomic	GCF_023060255.1	Brazil	0_0_124_0_0_6_0_0_0_0	*Klebsiella pneumoniae*
GCF_023060305.1_ASM2306030v1_genomic	GCF_023060305.1	Brazil	0_0_83_3_6_0_1_0_0_0	*Klebsiella pneumoniae*
GCF_023060355.1_ASM2306035v1_genomic	GCF_023060355.1	Brazil	0_0_80_8_0_0_0_0_0_0	*Klebsiella pneumoniae*
GCF_023060505.1_ASM2306050v1_genomic	GCF_023060505.1	Brazil	0_0_197_0_17_0_9_1_0_0	*Klebsiella pneumoniae*
GCF_023060995.1_ASM2306099v1_genomic	GCF_023060995.1	Brazil	0_0_105_6_0_0_17_0_99_0	*Klebsiella pneumoniae*
GCF_023061015.1_ASM2306101v1_genomic	GCF_023061015.1	Brazil	0_0_105_6_0_0_69_0_0_0	*Klebsiella pneumoniae*
GCF_023061095.1_ASM2306109v1_genomic	GCF_023061095.1	Brazil	0_0_105_0_0_0_1_21_0_0	*Klebsiella pneumoniae*
GCF_023221815.1_ASM2322181v1_genomic	GCF_023221815.1	Argentina	0_0_105_0_37_0_1_0_0_0	*Klebsiella pneumoniae*
GCF_023221835.1_ASM2322183v1_genomic	GCF_023221835.1	Argentina	0_0_105_0_37_0_1_0_0_0	*Klebsiella pneumoniae*
GCF_023221875.1_ASM2322187v1_genomic	GCF_023221875.1	Argentina	0_0_105_0_37_0_1_0_0_0	*Klebsiella pneumoniae*
GCF_023276595.1_ASM2327659v1_genomic	GCF_023276595.1	Brazil	0_0_369_0_0_0_0_295_1_0	*Klebsiella pneumoniae*
GCF_023276605.1_ASM2327660v1_genomic	GCF_023276605.1	Brazil	0_0_197_0_4_0_31_10_0_0	*Klebsiella pneumoniae*
GCF_023276615.1_ASM2327661v1_genomic	GCF_023276615.1	Brazil	0_0_0_0_2_0_0_0_39_0	*Klebsiella pneumoniae*
GCF_023554495.1_ASM2355449v1_genomic	GCF_023554495.1	Chile	0_0_88_5_0_0_0_0_0_0	*Klebsiella pneumoniae*
GCF_023572235.1_ASM2357223v1_genomic	GCF_023572235.1	Brazil	0_0_105_0_11_0_0_0_2_0	*Klebsiella pneumoniae*
GCF_023572325.1_ASM2357232v1_genomic	GCF_023572325.1	Brazil	0_0_413_1_3_0_0_0_1_0	*Klebsiella pneumoniae*
GCF_023572345.1_ASM2357234v1_genomic	GCF_023572345.1	Brazil	0_0_22_27_0_0_86_0_0_0	*Klebsiella pneumoniae*
GCF_023572385.1_ASM2357238v1_genomic	GCF_023572385.1	Brazil	0_0_105_0_0_0_13_0_0_0	*Klebsiella pneumoniae*
GCF_023572615.1_ASM2357261v1_genomic	GCF_023572615.1	Brazil	0_0_105_6_0_0_69_5_0_0	*Klebsiella pneumoniae*
GCF_024494945.1_ASM2449494v1_genomic	GCF_024494945.1	Peru	0_0_548_0_0_0_0_1_0_0	*Klebsiella pneumoniae*
GCF_024495065.1_ASM2449506v1_genomic	GCF_024495065.1	Peru	0_0_388_1_5_0_3_0_0_0	*Klebsiella pneumoniae*
GCF_024495515.1_ASM2449551v1_genomic	GCF_024495515.1	Peru	0_0_388_1_5_0_4_0_0_0	*Klebsiella pneumoniae*
GCF_024534275.1_ASM2453427v1_genomic	GCF_024534275.1	Argentina	0_0_105_0_9_0_0_28_3_0	*Klebsiella pneumoniae*
GCF_024534295.1_ASM2453429v1_genomic	GCF_024534295.1	Argentina	0_0_105_6_0_0_0_0_2_0	*Klebsiella pneumoniae*
GCF_024734055.1_ASM2473405v1_genomic	GCF_024734055.1	Argentina	0_0_105_0_11_0_0_24_0_0	*Klebsiella pneumoniae*
GCF_024742235.1_ASM2474223v1_genomic	GCF_024742235.1	Argentina	0_0_105_0_9_0_0_28_4_0	*Klebsiella pneumoniae*
GCF_024742255.1_ASM2474225v1_genomic	GCF_024742255.1	Argentina	0_0_105_0_9_0_0_28_5_0	*Klebsiella pneumoniae*
GCF_024813565.1_ASM2481356v1_genomic	GCF_024813565.1	Brazil	0_0_369_0_0_0_0_0_1_0	*Klebsiella pneumoniae*
GCF_024813735.1_ASM2481373v1_genomic	GCF_024813735.1	Brazil	0_0_105_0_3_0_1_0_43_0	*Klebsiella pneumoniae*
GCF_024813765.1_ASM2481376v1_genomic	GCF_024813765.1	Brazil	0_0_105_0_9_0_0_28_1_0	*Klebsiella pneumoniae*
GCF_024813785.1_ASM2481378v1_genomic	GCF_024813785.1	Brazil	0_0_105_6_0_0_69_0_17_0	*Klebsiella pneumoniae*
GCF_024813815.1_ASM2481381v1_genomic	GCF_024813815.1	Brazil	0_0_105_0_9_0_0_28_1_0	*Klebsiella pneumoniae*
GCF_024813855.1_ASM2481385v1_genomic	GCF_024813855.1	Brazil	0_0_105_1_1_1_4_4_0_0	*Klebsiella pneumoniae*
GCF_024813875.1_ASM2481387v1_genomic	GCF_024813875.1	Brazil	0_0_105_0_11_0_0_0_3_0	*Klebsiella pneumoniae*

GCF_024814275.1_ASM2481427v1_genomic	GCF_024814275.1	Brazil	0_0_0_0_84_0_0_0_0_0	*Klebsiella pneumoniae*
GCF_024919375.1_ASM2491937v1_genomic	GCF_024919375.1	Ecuador	0_0_98_0_5_0_0_0_12_0	*Klebsiella pneumoniae*
GCF_025395495.1_ASM2539549v1_genomic	GCF_025395495.1	Brazil	0_0_19_1_14_0_0_0_0_0	*Klebsiella pneumoniae*
GCF_025395535.1_ASM2539553v1_genomic	GCF_025395535.1	Brazil	0_0_237_1_0_0_0_0_1_0	*Klebsiella pneumoniae*
GCF_025399265.1_ASM2539926v1_genomic	GCF_025399265.1	Argentina	0_0_105_6_0_0_0_0_2_0	*Klebsiella pneumoniae*
GCF_025399355.1_ASM2539935v1_genomic	GCF_025399355.1	Argentina	0_0_105_0_37_0_0_0_5_0	*Klebsiella pneumoniae*
GCF_025399375.1_ASM2539937v1_genomic	GCF_025399375.1	Argentina	0_0_105_0_9_0_0_28_6_0	*Klebsiella pneumoniae*
GCF_025399395.1_ASM2539939v1_genomic	GCF_025399395.1	Argentina	0_0_105_0_0_0_26_0_0_0	*Klebsiella pneumoniae*
GCF_025399535.1_ASM2539953v1_genomic	GCF_025399535.1	Argentina	0_0_105_0_0_0_27_0_0_0	*Klebsiella pneumoniae*
GCF_025399555.1_ASM2539955v1_genomic	GCF_025399555.1	Argentina	0_0_105_0_37_0_0_0_6_0	*Klebsiella pneumoniae*
GCF_025818935.1_ASM2581893v1_genomic	GCF_025818935.1	Trinidad and Tobago	0_0_0_0_2_0_168_0_0_0	*Klebsiella pneumoniae*
GCF_025818955.1_ASM2581895v1_genomic	GCF_025818955.1	Trinidad and Tobago	0_0_253_0_0_0_0_1_0_0	*Klebsiella pneumoniae*
GCF_025819035.1_ASM2581903v1_genomic	GCF_025819035.1	Trinidad and Tobago	0_0_395_0_11_0_0_1_1_0	*Klebsiella pneumoniae*
GCF_025819055.1_ASM2581905v1_genomic	GCF_025819055.1	Trinidad and Tobago	0_0_105_0_3_0_1_0_36_0	*Klebsiella pneumoniae*
GCF_025819065.1_ASM2581906v1_genomic	GCF_025819065.1	Trinidad and Tobago	0_0_230_0_4_1_0_0_1_0	*Klebsiella pneumoniae*
GCF_025819155.1_ASM2581915v1_genomic	GCF_025819155.1	Trinidad and Tobago	0_0_369_0_0_0_0_0_7_0	*Klebsiella pneumoniae*
GCF_025819195.1_ASM2581919v1_genomic	GCF_025819195.1	Trinidad and Tobago	0_0_371_2_1_0_0_0_0_0	*Klebsiella pneumoniae*
GCF_025819235.1_ASM2581923v1_genomic	GCF_025819235.1	Trinidad and Tobago	0_0_0_0_2_0_0_240_0_0	*Klebsiella pneumoniae*
GCF_025819355.1_ASM2581935v1_genomic	GCF_025819355.1	Trinidad and Tobago	0_0_221_1_96_0_0_0_0_0	*Klebsiella pneumoniae*
GCF_026191785.1_ASM2619178v1_genomic	GCF_026191785.1	Brazil	0_0_105_0_0_0_1_22_0_0	*Klebsiella pneumoniae*
GCF_026191935.1_ASM2619193v1_genomic	GCF_026191935.1	Brazil	0_0_105_0_24_0_0_0_1_0	*Klebsiella pneumoniae*
GCF_026191975.1_ASM2619197v1_genomic	GCF_026191975.1	Brazil	0_0_105_0_3_0_28_1_3_0	*Klebsiella pneumoniae*
GCF_026222835.1_ASM2622283v1_genomic	GCF_026222835.1	Brazil	0_0_105_0_9_0_0_28_1_0	*Klebsiella pneumoniae*
GCF_026222865.1_ASM2622286v1_genomic	GCF_026222865.1	Brazil	0_0_410_0_11_0_0_0_0_0	*Klebsiella pneumoniae*
GCF_026223155.1_ASM2622315v1_genomic	GCF_026223155.1	Brazil	0_0_541_4_0_0_0_0_0_0	*Klebsiella pneumoniae*
GCF_026223235.1_ASM2622323v1_genomic	GCF_026223235.1	Brazil	0_0_105_0_3_0_16_1_0_0	*Klebsiella pneumoniae*
GCF_026223255.1_ASM2622325v1_genomic	GCF_026223255.1	Brazil	0_0_105_0_3_0_16_1_0_0	*Klebsiella pneumoniae*
GCF_026223265.1_ASM2622326v1_genomic	GCF_026223265.1	Brazil	0_0_0_0_2_0_75_0_0_0	*Klebsiella pneumoniae*
GCF_026620175.1_ASM2662017v1_genomic	GCF_026620175.1	Brazil	0_0_22_21_2_0_0_0_0_0	*Klebsiella pneumoniae*
GCF_027124715.1_ASM2712471v1_genomic	GCF_027124715.1	Brazil	0_0_105_0_0_0_1_23_0_0	*Klebsiella pneumoniae*
GCF_027890995.1_ASM2789099v1_genomic	GCF_027890995.1	Brazil	0_0_105_0_0_0_1_0_3_0	*Klebsiella pneumoniae*
GCF_027891035.1_ASM2789103v1_genomic	GCF_027891035.1	Brazil	0_0_105_0_0_0_1_0_4_0	*Klebsiella pneumoniae*
GCF_027945645.1_ASM2794564v1_genomic	GCF_027945645.1	Brazil	0_0_22_27_0_0_0_126_0_0	*Klebsiella pneumoniae*
GCF_028067495.1_ASM2806749v1_genomic	GCF_028067495.1	Trinidad and Tobago	0_0_105_0_9_0_0_0_0_0	*Klebsiella pneumoniae*

GCF_029076025.1_ASM2907602v1_genomic	GCF_029076025.1	Brazil	0_0_105_0_0_0_1_4_0_0	*Klebsiella pneumoniae*
GCF_029079265.1_ASM2907926v1_genomic	GCF_029079265.1	Brazil	0_0_105_0_11_0_0_0_4_0	*Klebsiella pneumoniae*
GCF_029079765.1_ASM2907976v1_genomic	GCF_029079765.1	Brazil	0_0_105_6_0_0_69_0_1_0	*Klebsiella pneumoniae*
GCF_029193615.1_ASM2919361v1_genomic	GCF_029193615.1	Chile	0_0_429_0_25_0_1_1_0_0	*Klebsiella pneumoniae*
GCF_928375135.1_BB1542_assembly_hybrid_genomic	GCF_928375135.1	Venezuela	0_0_107_0_1_9_3_0_1_0	*Klebsiella pneumoniae*
